# A Templating Approach to Controlling the Growth of Coevaporated Halide Perovskites

**DOI:** 10.1021/acsenergylett.3c01368

**Published:** 2023-09-01

**Authors:** Siyu Yan, Jay B. Patel, Jae Eun Lee, Karim A. Elmestekawy, Sinclair R. Ratnasingham, Qimu Yuan, Laura M. Herz, Nakita K. Noel, Michael B. Johnston

**Affiliations:** † Department of Physics, University of Oxford, Clarendon Laboratory, Parks Road, Oxford OX1 3PU, United Kingdom; ‡ Institute for Advanced Study, Technical University of Munich, Munich, Lichtenbergstrasse 2a, D-85748 Garching Germany

## Abstract

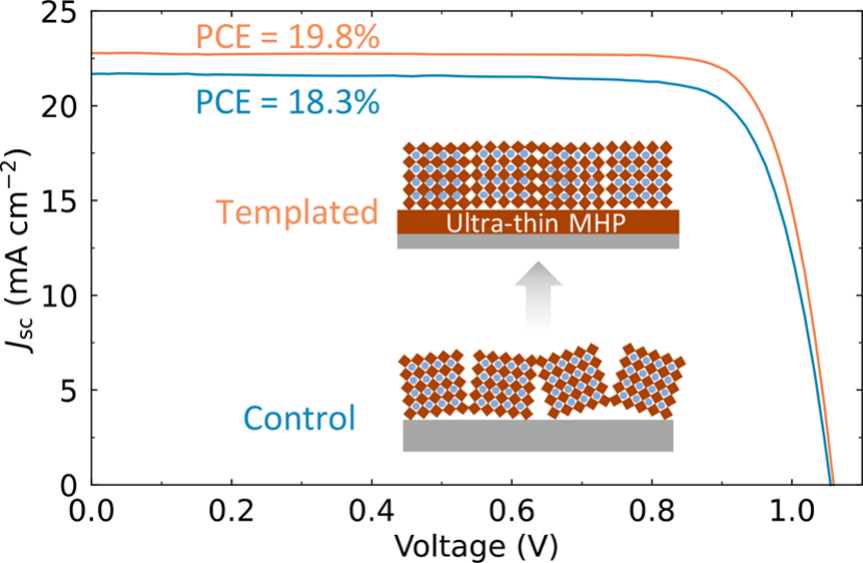

Metal halide perovskite semiconductors have shown significant potential for use in photovoltaic (PV) devices. While fabrication of perovskite thin films can be achieved through a variety of techniques, thermal vapor deposition is particularly promising, allowing for high-throughput fabrication. However, the ability to control the nucleation and growth of these materials, particularly at the charge-transport layer/perovskite interface, is critical to unlocking the full potential of vapor-deposited perovskite PV. In this study, we explore the use of a templating layer to control the growth of coevaporated perovskite films and find that such templating leads to highly oriented films with identical morphology, crystal structure, and optoelectronic properties independent of the underlying layers. Solar cells incorporating templated FA_0.9_Cs_0.1_PbI_3–*x*_Cl_*x*_ show marked improvements with steady-state power conversion efficiency over 19.8%. Our findings provide a straightforward and reproducible method of controlling the charge-transport layer/coevaporated perovskite interface, further clearing the path toward large-scale fabrication of efficient PV devices.

Metal halide perovskites (MHPs) have shown tremendous promise as absorber layers for the next-generation of photovoltaic (PV) devices, achieving certified power conversion efficiencies (PCEs) of up to 26% in single-junction solar cells and 33.7% in Si/perovskite tandems.^1^ This impressive device performance stems from a combination of factors: the desirable optoelectronic properties of MHP materials, including high absorption coefficients, long charge-carrier diffusion lengths and balanced charge-carrier transport;^2−4^ and concurrently, rapid improvements in fabrication approaches such as compositional engineering, tuning of crystallization kinetics, and interfacial engineering.^5−8^ Another interesting optoelectronic property of lead HPs is their “defect tolerance”, which means that bulk defects are located close to the bands and do not form deep charge-carrier traps.^9,10^ However, in a thin film, defect states often exist at the grain boundaries and interfaces where they can act as electronic traps, affecting charge-carrier transport in the films, hence hindering the performance of optoelectronic devices.^10−13^ Given that surface defects are influenced by various factors such as poor morphology and energy level misalignment, significant effort has been placed on these areas in order to improve optoelectronic device performance.^14−19^

To date, many of the key advances in MHP solar cell research have been made using solution-processed perovskite films. However, recent developments in dry, vapor deposition methods have resulted in devices with >24% PCE in single-junction architectures.^20−25^ Thermal vapor deposition allows for uniform, conformal coating of films and fine control of the thickness, affording significant opportunity to achieve high-throughput fabrication and large-scale production.^26−28^ However, previous research has highlighted the paramount importance of having a high-quality charge transport layer (CTL)/perovskite interface in order to unlock the full potential of vapor-deposited perovskites.^29−31^ Xu et al. have contended that a lead iodide (PbI_2_) phase located near the bottom of the perovskite film, closest to the substrate, is unavoidably formed on all substrates during the initial deposition process—even under a methylammonium iodide (MAI)-rich environment—since the sticking coefficient (defined as the ratio of atoms adsorbed to all atoms incident upon the surface) of MAI is small compared to that of PbI_2_.^32^ However, as opposed to the sole formation of PbI_2_, it is quite likely that a mixture of precursor materials/phases is present in this bottom region. Meanwhile, Patel et al. have uncovered that compared to phenyl-C_61_-butyric acid methyl ester (PCBM), coevaporated devices using compact titanium dioxide (c-TiO_2_) as the bottom electron transport layer (ETL) experience more severe hysteresis. This clearly indicates the extent to which the nature of the substrate impacts the quality of the perovskite at the bottom interface.^33^ It is apparent that growing coevaporated films on inorganic substrates is not trivial, and therefore, in most cases, perovskite films are coevaporated onto organic layers in order to seed better crystal growth.^21,24−26,29,30^ This may be one of the factors hindering the further performance improvement of coevaporated perovskite PV devices, considering that the best performing single-junction devices utilize tin oxide (SnO_2_) as the ETL.^20^ As previously mentioned, one possible explanation for this observation is that the initial stages of the perovskite growth may lead to the deposition of a layer of material that deviates from the target stoichiometry. Meanwhile, in situ photoelectron spectroscopy studies have shown that the electronic properties of the initial 2–3 nm of the perovskite are strongly affected by the nature of the substrate, giving rise to band bending and additional defect states.^34^ Subsequent investigations have revealed that the surface of the substrate material exerts a substantial impact beyond the bottom CTL/perovskite interface, leading to alterations in the morphology of the entire film.^35−37^ Consequently, achieving uniform deposition of alkylammonium halides and appropriate crystallization of the perovskite material is a critical requirement when selecting an appropriate CTL. Unfortunately, for vapor-deposited perovskites, this places significant constraints on the pool of viable CTLs. However, it should be noted that even when suitable substrates that meet the criteria above are identified, optimizing the evaporation parameters for deposition on various substrates remains a laborious task (as shown in Table S1). Furthermore, this issue limits the economic feasibility of industrial applications, as the most attractive scale-up technique is to produce all device layers on the same vacuum fabrication lines.^38^ Therefore, the ability to decouple the nucleation and growth of coevaporated perovskite films from the influence of substrate materials is of the utmost importance.

Here, we introduce an effective strategy to control reproducibly the growth of coevaporated perovskites by using a templating layer. We show that independent of the substrate choice for perovskite growth, the coevaporated perovskite films with the templating layer exhibit identical morphology, structure, and optoelectronic properties. Through a series of spectroscopic experiments, we present evidence that the optoelectronic properties of the perovskite remain unchanged after insertion of this templating layer. When these templated perovskite films are incorporated into devices, we observe improved device performance as a result of reduced interfacial resistance, (as indicated through increased short-circuit current (*J*_sc_) and fill factor (FF)). We attribute this improvement to accurate interface control, allowing for fine-tuning the stoichiometry of the initial perovskite deposited onto the substrate. Furthermore, the use of templating layers permits the fabrication of high-performance devices on both organic and inorganic CTLs in the same batch, which shows the universality of using templating layers in different device architectures. Overall, this templating strategy offers coevaporated perovskites more freedom in the selection of substrate materials and provides a way to exert fine control over the bottom interface in perovskite optoelectronic devices.

In this study, we fabricate perovskite films with the composition FA_0.9_Cs_0.1_PbI_3–*x*_Cl_*x*_, using the four-source, coevaporation of formamidinium iodide (FAI), CsI, PbI_2_ and PbCl_2_ under high vacuum (further details are given in the SI). We carefully monitor the initial coevaporation process by introducing one additional shuttered quartz crystal microbalance (QCM) in close proximity to the substrate position. Having a shuttered QCM allows us to record substrate rates at different points throughout the process. Therefore, by simultaneously opening the QCM and substrate shutters, we can record the rates only after starting deposition on the substrate. Here, we observe that it takes some time for the rate to stabilize on a bare shuttered QCM during the initial stages of the evaporation. This stabilization time increases when the shuttered QCMs are precoated with CTLs (full details are provided in SI, Figures S1, S2a,b). This finding confirms that depending on the type of substrate used, a number of different initial crystal growth processes can affect the incorporation of subsequent precursors and the continuity of crystal growth. This then inevitably influences how effective charge transport through the perovskite film is, and finally has a significant impact on device performance.^35^ One concern arising from this observation is the difficulty in individually assessing the quality of the bottom interface since the bulk perovskite is also unavoidably changed when different substrates are employed.

A potential solution to this problem is to utilize a templating layer which can regulate the crystallization and growth of perovskite films, such that the influence of the underlying substrate is obviated. Alkylammonium halides readily adsorb onto the surface of metal halides to form perovskites,^29,39^ while evaporating metal halides onto various substrates is reproducible owing to excellent adhesion. For the sequential, two-step deposition technique, the inorganic layer (PbI_2_) is deposited onto the substrate followed by either the solution or vapor-based deposition of the organic layer, after which the film is annealed.^40^ Although this process may suffer from issues arising from the diffusion of the organic layer,^41^ it presents an interesting approach in terms of using PbI_2_ as a templating layer. To examine this, we precoat PbI_2_ onto the shuttered QCM and indeed find that this effectively reduces the time taken for the readings on the QCM to stabilize, as we show in Figure S2c. However, the presence of PbI_2_ still might introduce nonstoichiometric perovskite near the bottom interface.

To overcome these issues, we develop a novel approach of depositing a templating layer consisting of a stoichiometric, ultrathin (15 nm) MHP which can fully cover the underlying layer (Figure S3) via a vapor-based, two-step sequential deposition approach. Specifically, using thermal vapor deposition, we coevaporate the inorganic precursors (PbI_2_, CsI, and PbCl_2_) to a total thickness of 9 nm and then deposit a 9-nm film of FAI on top. We then anneal this film at 135 °C for 2 min in pure N_2_ gas at atmospheric pressure to form the ultrathin-MHP templating layer (see SI for further experimental details). This templating layer then acts as a seed for the growth of fully coevapoated MHP layers.

In Figure S2c, we show that by precoating the shuttered QCM with this templating layer, we are able to obtain the shortest stabilization time. To verify that the use of this templating layer is able to totally circumvent the compositional variations found during the initial phases of growth on untemplated substrates, we also deposit it on a 2,2′,7,7′-tetra(N,N-di-p-tolyl)amino-9,9-spirobifluorene (spiroTTB) precoated shuttered QCM. Here, we find that the rate stabilizes almost immediately, pointing toward a significantly reduced likelihood of compositional variation at the CTL/perovskite interface (Figure S2d). As such, we proceed to investigate the effect of this templating layer on the properties of coevaporated perovskite films.

To probe the universality of the templating approach, we select 5 different CTLs which are often used in high performance PSCs: spiroTTB, nickel oxide (NiO_*x*_), tin oxide (SnO_2_), C_60_ and poly[bis(4-phenyl)(2,4,6-trimethylphenyl)amine] (PTAA). After the templating layers are deposited, the initial characterization results show that these templating layers have consistent morphology and structure on different CTLs (Figures S4–S6). The perovskite films are then simultaneously coevaporated onto all these substrates with or without a templating layer, named as “templated” or “control” films, respectively.

First, we investigate the influence of the templating layer on the morphology of perovskite films by using scanning electron microscopy (SEM) and present the data in Figure 1. From the top-view SEM, we see that both control (Figure 1a–e) and templated (Figure 1k–o) films form uniform, homogeneous films.^24,35^Figure 1a–e shows that control films deposited on the various substrates have significant morphological changes. This is particularly evident on the inorganic substrates, in this case, NiO_*x*_ and SnO_2_. Conversely, the morphology of templated films (Figure 1k–o) is very similar in all cases, suggesting that the crystallization dynamics are now independent of the substrate material. Lower magnification images confirm that this similarity holds across longer length scales (Figure S7). Cross-sectional SEM images, consistent with top-view SEM images, show significant differences in the vertical ordering of apparent grains (Figure 1f–j). Specifically, we observe that on spiroTTB and SnO_2_, control films appear to have columnar growth, whereas on NiO_*x*_ and PTAA, films appear to consist of small, randomly sized grains with noticeable gaps. On C_60_, control films are also composed of smaller crystallites of random orientations. Interestingly, when films are grown on the templating layer, in all cases, the crystals appear to have formed more columnar structures with fewer voids and visible boundaries (Figure 1p–t). This suggests more continuous grain growth and thus, the formation of higher quality perovskite thin films, specifically with regards to improved charge transport through the absorber. As previously mentioned, MHPs have been used as top cells in perovskite/Si tandems, achieving the highest performance of any double-junction monolithic tandem solar cell.^1^ It is worth noting that the most efficient perovskite/Si tandems are constructed using textured Si as the bottom cell. Depositing conformal perovskite layers on such substrates via spin-coating is nontrivial and as such, devices of this sort are often fabricated using one of three types of two-step depostion methods:either entirely vapor-based, hybrid vapor/solution-based, or entirely solution-based.^42,43^ With this in mind, we consider the feasibility of using our templating approach to control the deposition of coevaporated perovskite films on textured Si. Here, we deposit control and templated films on Si substrates with pyramid heights of more than 10 μm (Figure 1u–x). The results show that coevaporation can readily produce conformal, pinhole-free films on top of the pyramidal structures. However, as with the substrates previously investigated, the cross-sectional profile of control films grown on the textured Si shows multiple crystallites stacked on top of each other in the vertical direction, while templated films again form vertically continuous, columnar structures. These initial results show the likelihood of successfully transferring our templating approach to a variety of substrates.

**Figure 1:**
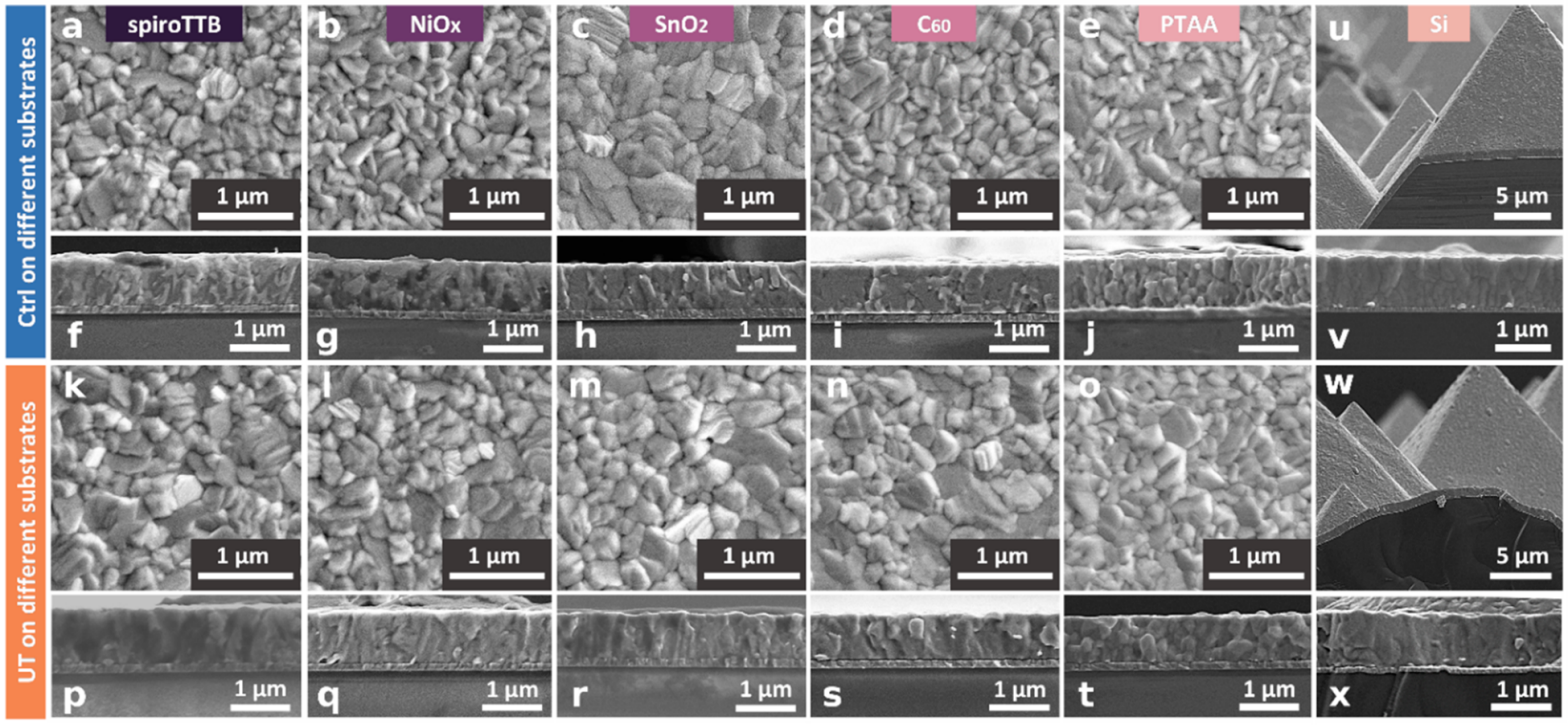
Morphological characterization of FA_0.9_Cs_0.1_PbI_3–*x*_Cl_*x*_ perovskite films deposited with and without an ultrathin MHP layer, labeled UT and Ctrl, respectively. Top-view scanning electron microscopy (SEM) images of Ctrl (a-e) and UT (k-o) films on spiroTTB, NiO_*x*_, SnO_2_, C_60_ and PTAA, respectively (see SI for lower magnification top-view SEM images). Cross-sectional SEM images of Ctrl (f–j) and UT (p–t) films on spiroTTB, NiO_*x*_, SnO_2_, C_60_ and PTAA. The cross-section profile of Ctrl (u and v) and UT (w and x) films on the textured Si (film thicknesses obtained from the cross-section SEM can be found in Table S2).

However, merely having a similar morphology is insufficient to confirm the utility of the templating layer. We now proceed to probe the structure and composition of the control and templated films. First, we measure the absorption spectra of these films and present the data in Figure S8 and Table S3. The absorption coefficient of a particular material should be consistent, as it is an intrinsic property. However, if the absorption coefficients of perovskite on different substrates are varied, this might suggest the presence of nonperovskite components in these films. Using the measured absorption spectra, we extract the bandgap values by fitting the absorption onsets with the Elliott model (details given in SI).^44^ Surprisingly, the bandgap values of control films grown on different substrates vary within the range of 1.57–1.60 eV. It is worth noting that these films were deposited during the same deposition run, and as such, their bandgaps should be identical, making a 30 meV difference in the bandgap significantly larger than one would expect. This lends credence to the hypothesis that variations in the composition of the first few nm of perovskite can indeed affect the stoichiometry of the bulk material.^34^ Encouragingly, however, we note that with the inclusion of a templating layer the absorption coefficient and bandgap of the deposited films (1.59 eV) are consistent, regardless of the nature of the underlying substrate. This suggests that with the templating layer, we are able to reproducibly control the crystallization and composition of the perovskite.

Next, we explore the degree of crystallinity of these films using X-ray diffraction (XRD). Figure 2a and b shows the XRD patterns of the control and templated films. Apart from the PbI_2_ peaks and ITO peaks, all other peaks in control and templated films can be assigned to the cubic perovskite phase (Pm3m) (Figures S9–S10).^28^ We note that the intensities of the perovskite (200) peaks in templated films are 2 to 10 times higher than those in the control films (Table S4). The significantly higher peak intensity, as well as smaller full width at half-maximum (fwhm) in templated films, suggests that perovskite films grown on this templating layer have a higher degree of crystallinity and are more highly oriented. To further probe the crystal orientation, we conducted grazing incidence wide-angle X-ray scattering (GIWAXS) measurements. In Figure 2c, we observe diffuse rings, representing many weak reflections across the entire arc, indicative of a polycrystalline film with many randomly oriented crystallites. Conversely, we clearly observe discrete spots in the diffraction pattern of templated films (Figure 2d), indicating a higher level of orientation. This change in orientation is depicted schematically in Figure 2e and f, respectively. While these measurements were carried out on quartz substrates, the trend holds on relevant CTLs such as spiroTTB (Figure S11).

**Figure 2:**
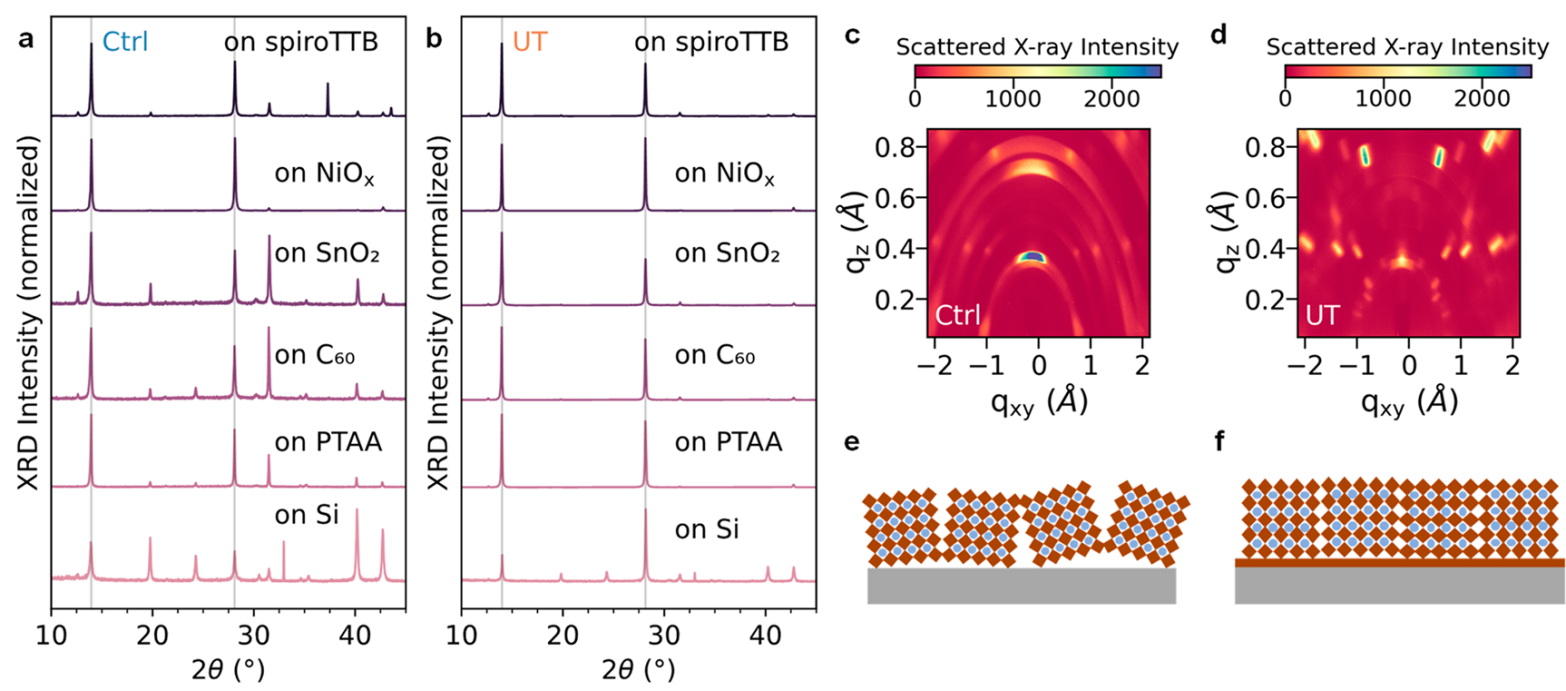
Structural characterization of FA_0.9_Cs_0.1_PbI_3–*x*_Cl_*x*_ perovskite films deposited with and without an ultrathin MHP layer, labeled UT and Ctrl, respectively. X-ray diffraction (XRD) patterns of Ctrl (a) and UT (b) films on spiroTTB, NiO_*x*_, SnO_2_, C_60_, PTAA and textured Si. Grazing incidence wide-angle X-ray scattering (GIWAXS) results of Ctrl (c) and UT (d) films deposited on z-cut quartz. Simplified schematic representations of randomly oriented Ctrl films (e) and highly oriented UT films (f).

Having established that when deposited on CTLs, the morphological and structural properties of control films are highly dependent on the nature of substrates and that the insertion of a templating layer circumvents these issues, we proceed to investigate the impact of this layer on the optoelectronic properties of the perovskite films. Figure S12a shows, in the absence of CTLs, a slightly increased absorption coefficient for templated films, while control and templated films have very similar absorption onsets at approximately 1.59 eV and overlapping photoluminescence (PL) emission. To assess the effect of the inclusion of a templating layer on the charge-carrier dynamics, we perform time-resolved terahertz photoconductivity spectroscopy. Control and templated films were probed using the optical-pump tetraherz-probe (OPTP) technique (Figure S12b).^45,46^ The bimolecular recombination constant *k*_2_ was extracted from the fluence-dependent transient photoconductivity decays (full details are provided in SI). Similar *k*_2_ values indicate almost unchanged electron–hole recombination in these two films. The corresponding charge-carrier mobilities (listed in Table S5) further show that the short-range mobilities are unaffected by the insertion of the templating layer. We then probe control and templated films via time-resolved PL (Figures S12c, S13 and Table S6) to extract the trap-assisted recombination (monomolecular recombination) *k*_1_,^47,48^ and we observe no significant difference between control (≈2.3 x10^6^ s^–1^) and templated (≈1.8 x10^6^ s^–1^) films.

Our results thus far show that the templating layer gives us better control over the crystallization and composition of vapor-deposited MHP films, while preserving their optoelectronic properties. To investigate the efficacy of this approach in solar cells, we incorporate these films into an all-vacuum-deposited, p-i-n device with the structure ITO/spiroTTB/control or templated MHP films/C_60_/Bathocuproine (BCP)/Ag (as shown in Figure 3a). After the optimization of the thickness of the perovskite layer (Figure S14), we show the best performing devices using control and templated films devices in Figure 3b and c, and list the performance parameters in Table 1. The statistical results shown in Figure 3a are taken from a total of 16 devices across 3 different batches and show that devices made with the templating layer have a narrower distribution in performance than control devices, indicating better reproducibility. Interestingly, the insertion of the templating layer results in improved device performance, boosting the PCE from 18.3% (18.2% stabilized power output (SPO)) to 19.3% (19.1% SPO), as shown in Figure 3c. This improvement is a result of the increased *J*_sc_ and FF in templated devices. The integrated *J*_sc_ values obtained from the corresponding external quantum efficiency (EQE) (Figure 3d) are 22.2 mA cm^–2^ and 23.9 mA cm^–2^ for control and templated devices, respectively, which are in close agreement with the measured values. Additionally, Table 1 illustrates the discrepancy of average SPO between templated (18.3 ± 0.1%) and control (16.9 ± 0.3%) devices, confirming the efficiency enhancement in templated devices (the statistical results of SPO and steady-state current density are given in Figure S15). We further investigate the operational stability of unencapsulated control and templated devices aged at 85 °C in a dark N_2_ atmosphere. The average SPOs are shown in Figure S16. The measured lifetimes for 80% of the peak SPO (T_80 ave_) of control and templated devices are 408 and 480 h, respectively. The slightly prolonged lifetime of the templated devices can be ascribed to the slower degradation of the templated perovskite films (Figure S17).

**Figure 3:**
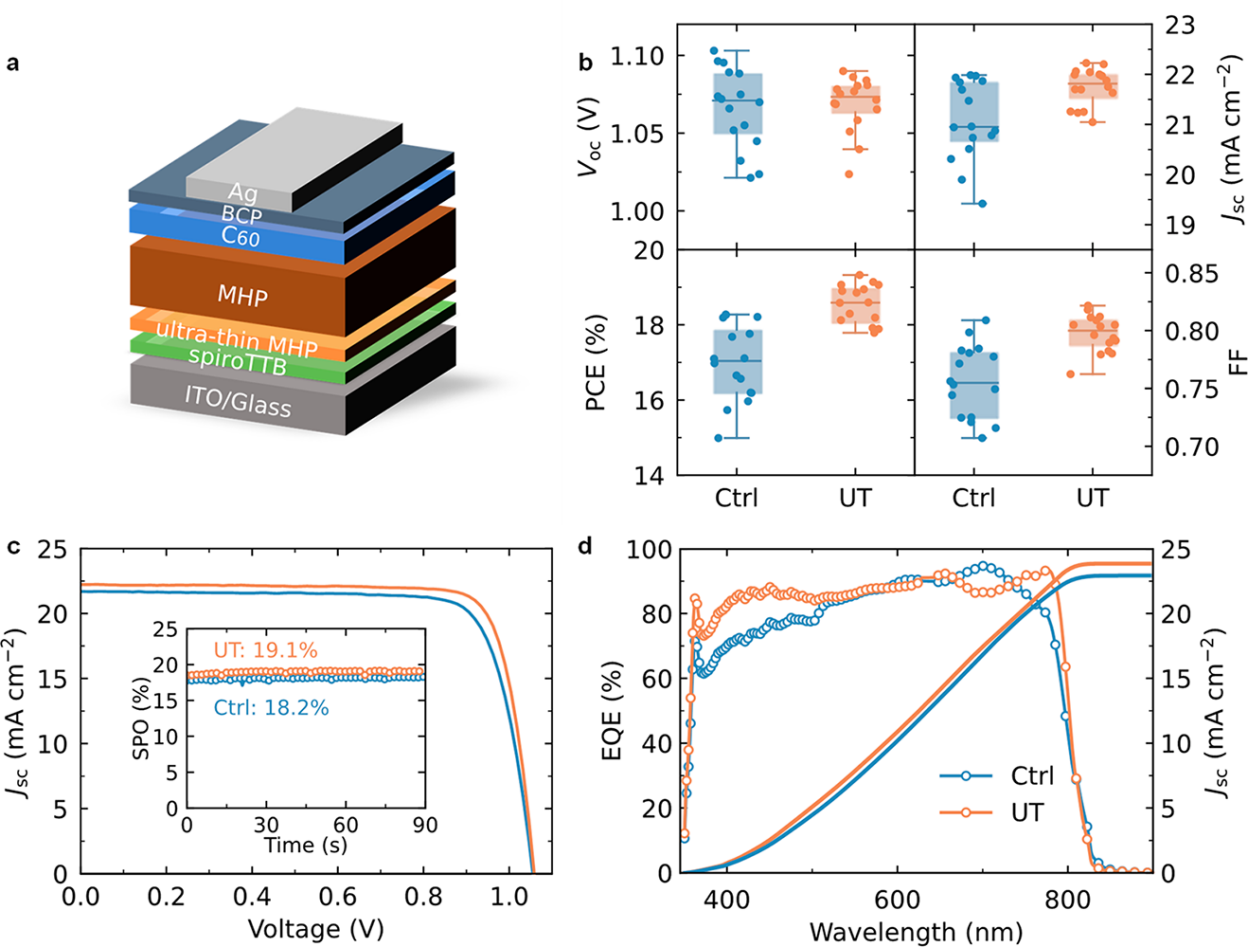
Improvement of device performance. The absorber layers are FA_0.9_Cs_0.1_PbI_3–*x*_Cl_*x*_ perovskite films with and without an ultrathin MHP layer, labeled UT and Ctrl, respectively. (a) Statistical results of Ctrl and UT device parameters. (b) *J–V* characteristics and corresponding SPO (inset) for the champion cell using the Ctrl and UT films. (c) EQE, and integrated *J*_SC_ for the devices. The integrated *J*_SC_ values for the Ctrl and UT devices are 22.2 and 23.9 mA·cm^–2^, respectively.

**Table 1:** Champion and Average Device Performance Parameters for Solar Cells Fabricated from FA_0.9_Cs_0.1_PbI_3–*x*_Cl_*x*_ Perovskite Films with and without an Ultrathin MHP Layer, Labeled UT and Ctrl, Respectively^a^

Device	*J*_sc_ (mA cm^–2^)	*V*_**oc**_ (V)	PCE (%)	FF	SPO (%)	*R*_sh_ (kΩ)	*R*_s_ (Ω)
Ctrl (champion)	21.7	1.06	18.3	0.80	18.2	17.6	10.3
Ctrl (average)	21.1 ± 0.2	1.07 ± 0.02	17.0 ± 0.3	0.76 ± 0.04	16.9 ± 0.3	9.1 ± 3.1	11.2 ± 1.3
UT (champion)	22.2	1.06	19.3	0.82	19.1	15.0	8.4
UT (average)	21.8 ± 0.1	1.07 ± 0.02	18.5 ± 0.1	0.80 ± 0.02	18.3 ± 0.1	14.0 ± 0.7	8.4 ± 0.1

a The values given in this table represent scans from open-circuit to short-circuit conditions.

By fitting the tail of the EQE spectrum, we obtain the Urbach energy (*E*_u_), which is a measure of the degree of electronic disorder in semiconductor films.^49,50^ The results (Figures S18–19) show that the *E*_u_ values are nominally the same (14.0 ± 0.1 and 13.9 ± 0.1 meV for control and templated films, respectively), implying similar electronic disorder in both films, in line with both the OPTP results and *V*_oc_ values of control and templated devices. Given that the performance improvement is clearly not obtained through altering intrinsic material properties, we re-examine the *J–V* curves to gather more information about the device stacks. Table 1 shows the average shunt resistance (*R*_sh_) and series resistance (*R*_s_). Compared with control devices, in templated devices, the average *R*_s_ is reduced from 11.2 ± 1.3 Ω to 8.4 ± 0.1 Ω, and the average *R*_sh_ increases from 9.1 ± 3.1 kΩ to 14.0 ± 0.8 kΩ. The reduced average *R*_s_ for the templated devices may be ascribed to the absence of a PbI_2_-rich layer at the bottom interface.^32^ This would also explain the increase in *J*_sc_ as the reduced resistance at the interface would allow for the more efficient extraction of charges. In addition, as low *R*_sh_ values are usually linked with pinholes and uneven perovskite films,^51,52^ the improved *R*_sh_ in templated devices may be attributed to the formation of higher-quality films comprised of columnar crystallites which have fewer boundaries in the direction of charge transport, and a more uniform distribution of the perovskite at the bottom interface.

Finally, we show that our templated growth approach is relevant for a wide range of solar-cell device architectures, not just p-i-n devices utilizing spiroTTB. We deposited control and templated films on organic (PTAA and C_60_) and inorganic (SnO_2_) substrates to fabricate both p-i-n and n-i-p devices, as shown in Figure 4a-f. In all cases, device performance is markedly improved when the templating layer is inserted, which we attribute to the increased *J*_sc_ and FF in these devices (Figures S20–S21 and Table S7). Furthermore, by growing the templated films on spiroTTB, SnO_2_, and C_60_ substrates, p-i-n and n-i-p devices can be fabricated in one deposition run. This process was previously nontrivial as deposition conditions needed to be optimized for specific substrates. The *J–V* characteristics of champion spiroTTB/templated-MHP, SnO_2_/templated-MHP and C_60_/templated-MHP devices shown in Figure 4g illustrate that the *J*_sc_ values in these three-types of devices are comparable. The small difference in *V*_oc_ can be explained by the slightly different energetic alignments between perovskite and various CTLs. We show the statistical SPO and PCE results in Figure 4h and 4i (more statistics of the devices are given in Figure S22). For the best-performing spiroTTB/templated-MHP, SnO_2_/templated-MHP and C_60_/templated-MHP devices, the PCEs were 19.8% (19.8% SPO), 19.0% (17.8% SPO) and 18.8% (18.6% SPO), respectively (Table S8). The integrated *J*_sc_ values obtained from the EQE data in Figure 4j are consistent with those of the *J–V* measurements. This finding confirms that the use of perovskite templating layers allows for the fabrication of high performance p-i-n and n-i-p devices in the same batch, as a result of providing an effective and reproducible route of improving the quality of the CTL/perovskite interface and exerting fine control over the composition and crystallization dynamics of vapor-deposited perovskite films. Furthermore, this templating method is not limited to perovskite films with a composition of FA_0.9_Cs_0.1_PbI_3–*x*_Cl_*x*_. We observe a similar improvement in device performance for FA_0.9_Cs_0.1_PbI_3_-based devices (Figure S23), indicating the universality of the templating layer to different perovskite compositions.

**Figure 4:**
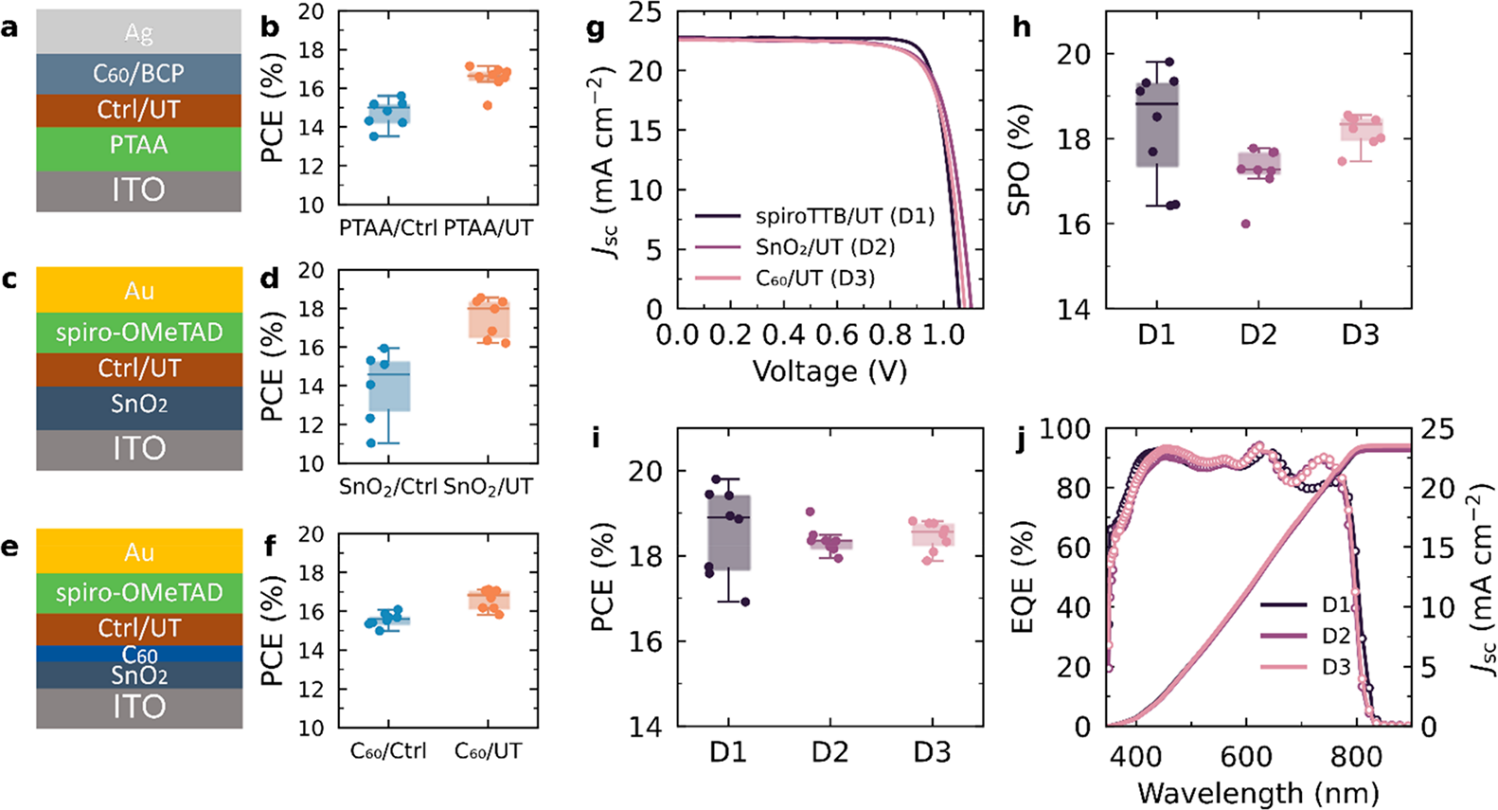
Impact of the ultrathin MHP layer on different charge transport layers. The absorber layers are FA_0.9_Cs_0.1_PbI_3–*x*_Cl_*x*_ perovskite films with and without an ultrathin MHP layer, labeled UT and Ctrl, respectively. Schematics for the p-i-n device architecture (a) and n-i-p device architectures with SnO_2_ (c) and C_60_ (e) n-type layers, with the corresponding statistical PCE results of Ctrl and UT devices (b, d, f). *J–V* characteristics (g), statistical SPO (h), statistical PCE (i), EQE, and integrated *J*_SC_ (j) for the champion p-i-n and n-i-p UT devices made in the same batch.

In conclusion, we have successfully established a method of controlling the buried interface in vapor-deposited perovskite films and have decoupled the nucleation and growth of these films from the influence of substrate materials. By inserting a templating layer between the perovskite film and the substrate, we are able to form highly oriented coevaporated perovskite films with identical morphology, structure, and optoelectronic properties on a variety of different materials. The inclusion of this templating layer results in improved solar-cell device performance, as a result of reduced interfacial resistance (increased *J*_sc_ and FF). We attribute this improvement to our ability to exert fine control of the composition of the initial perovskite deposited onto the substrate. These results provide an effective and reproducible method for controlling the buried charge transport layer/perovskite interface in vapor-deposited perovskite solar cells, further increasing the competitiveness of this deposition technique, moving the field closer to large-scale fabrication of a wide-range of efficient perovskite optoelectronic devices.

## Supporting Information Available

Additional details about experimental methods, SEM morphology, XRD, GIWAXS, OPTP spectra, and device analysis (PDF)
