# Correction to “A Templating Approach to Controlling the Growth of Coevaporated Halide Perovskites”

**DOI:** 10.1021/acsenergylett.3c02121

**Published:** 2023-10-18

**Authors:** Siyu Yan, Jay B. Patel, Jae Eun Lee, Karim A. Elmestekawy, Sinclair R. Ratnasingham, Qimu Yuan, Laura M. Herz, Nakita K. Noel, Michael B. Johnston

Page 4011. The axes of the GIWAXS figures in Figure 2c,d were labeled incorrectly. The corrected figure is shown below.

Supporting Information. Figure S11a,b, in which Figure 2 panels c and d are replotted for easier visualization, is also corrected.

**Figure 2:**
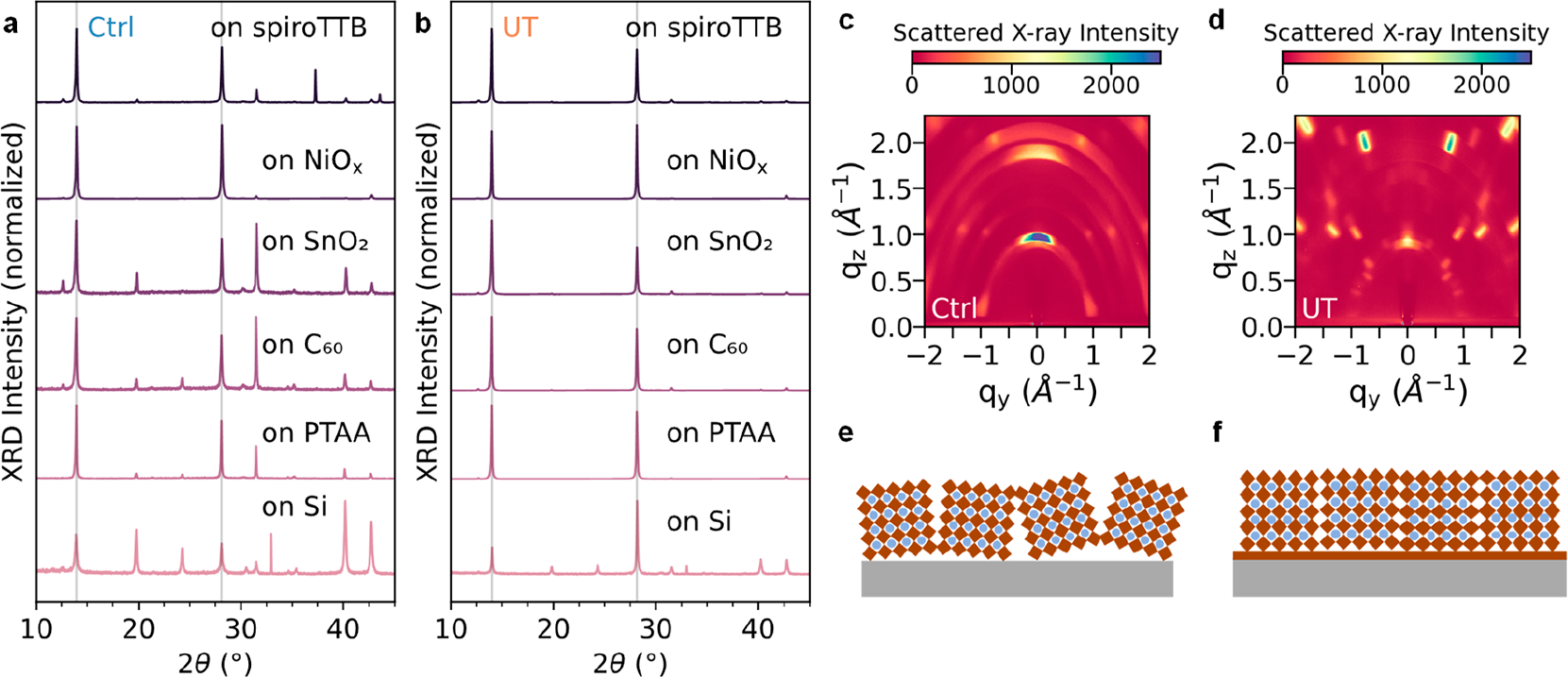
Structural characterization of FA_0.9_Cs_0.1_PbI_3–*x*_Cl_*x*_ perovskite films deposited with and without an ultrathin MHP layer, labeled UT and Ctrl, respectively. X-ray diffraction (XRD) patterns of Ctrl (a) and UT (b) films on spiroTTB, NiO_*x*_, SnO_2_, C_60_, PTAA and textured Si. Grazing incidence wide-angle X-ray scattering (GIWAXS) results of Ctrl (c) and UT (d) films deposited on z-cut quartz. Simplified schematic representations of randomly oriented Ctrl films (e) and highly oriented UT films (f).

## Supporting Information Available

Additional details about experimental methods, SEM morphology, XRD, GIWAXS, OPTP spectra, and device analysis (corrected) (PDF)

