# Supplementary material for: Correction to “A Templating Approach to Controlling the Growth of Coevaporated Halide Perovskites”
Source: ACS Energy Lett. 2023 Oct 18;8(11):4714–5. doi: 10.1021/acsenergylett.3c02121 (PMC10644385; doi:10.1021/acsenergylett.3c02121)
Supplement: Supplementary file 1 — nz3c02121_si_001.pdf [file nz3c02121_si_001.pdf]

Supplementary Information

for

A Templating Approach to Controlling the Growth of Co-evaporated Halide Perovskites

Siyu Yan,[a] Jay B. Patel,[a] Jae Eun Lee,[a] Karim A. Elmestekawy,[a] Sinclair R. Ratnasingham,[a] Qimu Yuan,[a] Laura M. Herz,[a,b] Nakita K. Noel[a] and Michael B. Johnston*[a]*

[a] Department of Physics, University of Oxford, Clarendon Laboratory, Parks Road, OX1 3PU, UK

[b] Institute for Advanced Study, Technical University of Munich, Munich, Lichtenbergstrasse 2a, D-85748 Garching, Germany

E-mail: michael.johnston@physics.ox.ac.uk; nakita.noel@physics.ox.ac.uk

Table of Contents

Experimental Section 3

1. Materials and Chemicals 3
2. Perovskite and Film Fabrication 3
   - Substrate Cleaning 3
   - Hole Transport Layers 3
   - Electron Transport Layers 3
   - Ultrathin Perovskite Thin Films (ultra-thin MHP) 3
   - Perovskite Thin Films 3
   - Electrodes 4
3. Scanning Electron Microscopy 4
4. X-ray Diffraction Measurements 4
5. Grazing Incidence Wide Angle X-ray Scattering Measurements 4
6. Absorption Spectra 4
   - Fourier Transform Infrared Measurements 4
   - Elliott Fitting 4
7. Photoluminescence Measurements 5
   - Steady-State Photoluminescence Measurements 5
   - Time-Resolved Photoluminescence Measurements 5
   - Trap-Assisted Recombination Fitting 5
8. Optical-Pump-Teraherz-Probe Spectroscopy 6
   - Mobility Fitting 6
   - K_2 obtained from fits to THz photoconductivity transients 7
9. Device Measurements 7
   - Current Density-Voltage Characteristics 7
   - External Quantum Efficiency 7
   - Urbach Energy 8

Supporting Data and Discussion 9

1. Evaporation Parameters 9
2. Characterization of the templating layers 13
3. Film Morphology 14
4. Absorption Measurements 15
5. Crystallinity and Orientation Characterization 17
6. Recombination Studies in Films 20
7. Device Optimization and Analysis 23

References 31

Experimental Section

1. Materials and Chemicals

Lead iodide (PbI_2, 99.999%, metal basis), cesium iodide (CsI, 99.99%), and lead chloride ($PbCl_2$, 99.998% metal basis) were purchased from Alfa-Aesar. Formamidinium iodide (FAI) was purchased from Greatcell Solar. Fullerene (C_{60}, 99.9%) was purchased from Acros Organics. Bathocuproine (BCP) (99.5%) was purchased from Sigma-Aldrich. Poly[bis(4-phenyl)(2,4,6-trimethylphenyl)amine (PTAA) was purchased from Xi'an Polymer Light Technology. Unless stated otherwise, all other materials and solvents were purchased from Sigma-Aldrich. In this work, all materials were used as received without further purification.

2. Perovskite and Film Fabrication

Substrate Cleaning

Indium-doped tin oxide (ITO) coated glass (Biotain, 30x30x1.1 mm, 10~15 ohm/sq) was used as the substrate. The substrates were cleaned in a series of ultrasonic baths using industrial detergent Decon90 (1% vol in deionized water), deionized water, acetone, and isopropyl alcohol (each step for 5 minutes). After ultrasonic cleaning, the substrates were dried with nitrogen gas, and the substrates were then placed in the UV-Ozone for 10 minutes.

Hole Transport Layers

SpiroTTB was vacuum-deposited in the thermal evaporator chamber to 5 nm at 0.1 Å/s. The rate was controlled using a gold-plated quartz crystal microbalance (QCM). The as-prepared substrate was ready to use.

PTAA was dissolved in toluene with the concentration of 2 mg/mL. 100 uL solution was dynamically deposited onto a substrate at a speed of 6000 rpm for 30 s. After spin-coating, the substrate was annealed at 100 ^{o}C for 10 minutes. After cooling down to room temperature, the substrate was ready to use.

Spiro-OMeTAD (Lumtec) was dissolved in Chlorobenzene at a concentration of 85 mg/mL, with added lithium bis(trifluoromethanesulfonyl)imide (Li-TFSI) dissolved in 1-Butanol at a concentration of 170 mg/mL, and tert-butylpiridine (tBP) at a concentration of 33 µL/mL. The solution was then spin-coated dynamically at 2000 rpm for 45 s. Before depositing the final electrode, the films were kept in a desiccator overnight to oxidise the spiro-OMeTAD.

Electron Transport Layers

C_{60} was vacuum-deposited in the thermal evaporator chamber to 25 nm at 0.1 Å/s. BCP was vacuum deposited in the thermal evaporator chamber to 5 nm at 0.1 Å/s. The as-prepared substrate was ready to use.

200 µL SnO_2 colloidal solution (diluted to 2.5 weight %) was deposited by static spin-coating at 3000 rpm for 30 s (1000 rpm ramp), followed by annealing at 150°C for 30 min in ambient air.

Ultrathin Perovskite Thin Films (ultra-thin MHP)

PbI_2, CsI, and $PbCl_2$ were co-evaporated with the molar ratio of CsI: PbI_2: $PbCl_2$ = 0.1: 0.8: 0.39 in a custom-built thermal evaporator chamber to deposit 9 nm films. Without breaking the vacuum, 9 nm FAI was evaporated on top of inorganic films. All ultrathin-MHP samples were annealed in an N_2 glovebox at 135 °C for 2 minutes. After cooling down to room temperature, ultra-thin MHP films were ready to use.

Perovskite Thin Films

FAI, PbI_2, CsI, and $PbCl_2$ were co-evaporated with the molar ratio of FAI: CsI: PbI_2: $PbCl_2$ = 0.9: 0.1: 0.8: 0.39 in a custom-built thermal evaporator chamber to deposit 630 nm films with the final compositions of $FA_{0.9}Cs_{0.1}PbI_{3-x}Cl_x$. During the evaporation, the pressure was typically < 5 × 10^{-6} mbar. The source rates were kept constant using gold-plated quartz microbalances (QCM) and PID-loop-control software.

The sublimation rate of the precursors was controlled using QCMs adjacent to the crucible. Unless specified otherwise, all samples were annealed in an N_2 glovebox at 150 °C for 5 minutes and at 135 °C for 25 minutes. After cooling down to room temperature, films were ready to use. We evaporated $PbCl_2$ with the molar ratio of 39%, while kept PbI_2 at 80% of the molar ratio.

Electrodes

100nm silver (Ag) contacts were thermally evaporated with shadow masks under high vacuum (<$6x10^{-6}$ torr) using a thermal evaporator (Nano 36, Kurt J. Lesker) placed in ambient environment.

3. Scanning Electron Microscopy

Scanning electron microscopy (SEM) images were taken by a Hitachi S-4300 microscope using the secondary electron detection mode, with 3 kV accelerating voltage and 10 µA emission current. The thicknesses of the perovskite films shown in Table S2 were calculated by the mean thickness of 3 measurements across the cross-section SEM in Figure 1.

4. X-ray Diffraction Measurements

X-ray diffraction measurements (XRD) were measured using a Panalytical X-pert powder diffractometer. The Cu-Kα X-ray source (λ=1.54 Å) was set to 40 kV voltage and 40 mA current. The XRD patterns were corrected by shifting the 2θ-axis based on the ITO substrate reference peak at 2θ=30.3^{o}. HighScore Plus software was used to implement Rietveld refinement to extract lattice parameters.

5. Grazing Incidence Wide Angle X-ray Scattering Measurements

We performed the grazing incidence wide angle X-ray scattering (GIWAXS) measurements using a Rigaku Smartlab X-ray diffractometer using the Cu-Kα X-ray source and a HyPix-3000 2D X-ray detector in a Bragg-Brentano reflection geometry.

6. Absorption Spectra

Fourier Transform Infrared Measurements

Absorption spectra were carried out using a Bruker Vertex 80v Fourier-Transform Infrared (FTIR) spectrometer with a near-infrared source, calcium fluoride beam splitter and a silicon diode detector. ITO or quartz substrates were used as reference substrates here. The absorption coefficient (α) was calculated using the following formula

$$\alpha = \frac{1}{d} log_{10}\left(\frac{1-R}{T}\right), \quad \text{(Eq-S1)}$$

where R is the reflectance spectrum, T is the transmittance spectrum, and d is the film thickness.

Elliott Fitting

The absorption coefficient spectra were fitted by the Elliott's model to obtain the optical bandgap (E_g), exciton binding energy (E_b) and electronic sub-bandgap disorder (γ). The Elliott's model represents the total absorption coefficient ($\alpha(E)$) as a linear combination of the absorption coefficient from bound excitons ($\alpha_{EX}(E)$) and electron-hole continuum states ($\alpha_C(E)$) for a direct semiconductor as shown below[1, 2]

$$\alpha(E) = \alpha_{EX}(E) + \alpha_C(E), \quad \text{(Eq-S2)}$$

The contribution from $\alpha_{EX}(E)$ is expressed as

$$\alpha_{EX}(E) = \frac{b_0}{E}\sum_{n=1}^{\infty}\frac{4\pi E_b^{3/2}}{n^3}\delta\left(E - \left[E_g - \frac{E_b}{n^2}\right]\right), \quad \text{(Eq-S3)}$$

where b_0 is a constant of proportionality that includes the electric dipole transition matrix element between the valence and conduction band, $|\langle\Psi_c|P|\Psi_v\rangle|^2$ and n is a positive integer quantum number. Eq-S3 describes a lines series at energies $-E_b/n^2$ below E_g and their magnitudes are proportional to $-1/n^3$. The contribution from $\alpha_C(E)$ is expressed as

$$\alpha_C(E) = \frac{b_0}{E}\left[\frac{2\pi\sqrt{\frac{E_g}{E-E_g}}}{1-exp\left(-2\pi\sqrt{\frac{E_g}{E-E_g}}\right)}\right]c_0^{-1}JDoS(E), \quad \text{(Eq-S4)}$$

where the joint density of states ($JDoS$) is given by

$$JDoS(E) = \begin{cases} c_0\sqrt{E-E_g}, & for\ E > E_g \\ 0, & otherwise \end{cases}, \quad \text{(Eq-S5)}$$

and the joint density of states constant (c_0) is given by

$$c_0 = \frac{1}{(2\pi)^2}\left(\frac{2\mu}{\hbar^2}\right)^{3/2} \times 2, \quad \text{(Eq-S6)}$$

where μ is the reduced effective mass of the electron-hole system, which is assumed to be 0.15 m_e.[3] The term in the square brackets in Eq-S4 is the Coulombic enhancement factor (ξ), which represents the probability of an electron and a hole existing in the same space. The second term $c_0^{-1}JDoS(E)$ represents the absorption coefficient of free electrons and holes in the absence of Coulombic attraction, $\alpha_{Free}(E)$.

The linear combination of the contributions from the excitonic and continuum states as shown in Eq-S2 is then convolved with a broadening function, which is written as

$$g(E) = \frac{1}{\cosh\frac{E-E_X}{\gamma}}, \quad \text{(Eq-S7)}$$

where γ is assigned as the electronic sub-bandgap disorder, $E_x = E_g$ when convolved with $\alpha_C(E)$,and E_x is equal to the line series when convolved with $\alpha_{EX}(E)$. It is worth noting that γ is also attributed to an electron-phonon coupling in other literature but with the absence of temperature dependence measurements, we mainly attribute the broadening to the degree of disorder.[1, 4]

7. Photoluminescence Measurements

Steady-State Photoluminescence Measurements

A 470 nm diode laser (PicoHarp, LDH-D-C-470M) was used to illuminate the samples, on a continuous wave with the intensity of 160 mW/cm^2. The PL emitted by the samples was coupled into a grating spectrometer (Princeton Instruments, SP-2558) and then directed onto a silicon iCCD (PI-MAX4, Princeton Instruments). The samples were measured under air atmosphere.

Time-Resolved Photoluminescence Measurements

A 470 nm picosecond pulsed diode laser (PicoHarp, LDH-D-C-470M) operated at 1MHz, with the fluence of 60 nJ/cm^2 was used to measure the samples in air. The PL emitted by the samples was collected by a photon-counting detector (PDM series from MPD) and the timing was controlled using a PicoHarp300 event timer.

Trap-Assisted Recombination Fitting

The photoluminescence decays for $FA_{0.9}Cs_{0.1}PbI_{3-x}Cl_x$ perovskite films deposited with and without an ultra-thin MHP layer, labelled UT and Ctrl respectively, were fitted using a stretched exponential that can be expressed in the form of

$$I(t) = I_0 e^{-(\frac{t}{\tau_c})^\beta},^5 \quad \text{(Eq-S8)}$$

where $I(t)$ is the time dependent PL intensity, I_0 is the initial PL intensity, t is time, τ_c is the characteristic lifetime, and β is the distribution parameter. Based on the characteristic lifetime and distribution parameter, we can calculate the average lifetime τ_{avg}, in the form of

$$\tau_{avg} = \frac{\tau_c}{\beta}\Gamma(\frac{1}{\beta}), \quad \text{(Eq-S9)}$$

$$\Gamma\left(\frac{1}{\beta}\right) = \int_0^{\infty} x^{(1-\beta)/\beta} e^{-x} dx, \quad \text{(Eq-S10)}$$

and the monomolecular recombination rate k_1 can be calculated by the following equation

$$k_1 = \frac{1}{2\tau_{avg}},^{6} \quad \text{(Eq-S11)}$$

8. Optical-Pump-Teraherz-Probe Spectroscopy

The optical-pump-terahertz-probe (OPTP) spectroscopy was taken to measure the mobility and bi-molecular recombination rate in Ctrl and UT films on z-cut quartz. An amplified laser system (Spectra Physics, MaiTai-Empower-Spotfire) with a central wavelength 800 nm, 35 fs pulse duration and 5kHz repetition rate was split into THz beam, pump beam and gate beam. The THz radiation was generated by a spintronic emitter based on inverse spin hall effect. The electro-optic sampling with a 1mm-thick ZnTe (110) crystal, a Wollaston prism and a pair of balanced photodiodes were used to detect the pulse. The THz pulse was measured in transmission geometry. The pump beam was generated at 400 nm by a β-barium-borate (BBO) crystal. The samples were photoexcited as various fluences ranging from 2.38 to 38.75 μJ/cm^2 under vacuum (<10^{-2} mbar).

Mobility Fitting

The effective charge-carrier mobility was extracted from the OPTP data using the method illustrated previously by Wehrenfennig *et al.*[7] The sheet photoconductivity ΔS of a thin film between two media of refractive indices n_A and n_B with its thickness much smaller than the THz wavelength can be expressed as

$$\Delta S = -\epsilon_0 c(n_A + n_B)\left(\frac{\Delta T}{T}\right), \quad \text{(Eq-S12)}$$

where ϵ_0 is the vacuum permittivity, c is the speed of light and $\Delta T/T$ is the ratio of photo-induced change in the THz electric field to the transmitted THz electric field in the dark. In this study, n_A is the refractive index of a z-cut quartz, which is 2.13 and n_B is the refractive index of vacuum which is 1.

Deriving the charge-carrier mobility μ from ΔS requires the knowledge of the number of photo-excited charge-carriers N, which can be calculated as

$$N = \varphi\frac{E\lambda}{hc}\left(1 - R_{pump} - T_{pump}\right), \quad \text{(Eq-S13)}$$

where φ is the photon-to-charge branching ratio, E is the energy obtained in an optical excitation pulse with wavelength λ, h is the Planck's constant, R_{pump} and T_{pump} are the reflected and transmitted fractions of the pump beam. Then, μ can be calculated with

$$\mu = \frac{\Delta S A_{eff}}{Ne}, \quad \text{(Eq-S14)}$$

where A_{eff} is the effective area of the overlap of optical pump and THz probe pulse considering the Gaussian beam profiles and e is the elementary charge. By using the above equations, the effective mobility $\tilde{\mu} = \varphi\mu$ is expressed as

$$\varphi\mu = -\epsilon_0 c(n_A + n_B)\frac{A_{eff}hc}{E\lambda e\left(1-R_{pump}-T_{pump}\right)}\left(\frac{\Delta T}{T}\right), \quad \text{(Eq-S15)}$$

In this work, we assume $\varphi = 1$ given that the exciton binding energies are low and free charge-carriers are mostly generated right after excitation.[8] The charge-carrier mobility obtained here is the sum of electron and hole mobilities and they cannot be separated.

To obtain $\varphi\mu$, $\Delta T/T$ at time zero against E was plotted and a linear function was fitted based on Eq-S15 up to the point where nonlinear processes were not observed. Then, $\varphi\mu$ was obtained from the gradient.

<u>K_2 obtained from fits to THz photoconductivity transients</u>
The recombination dynamics in a perovskite semiconductor may be governed by first, second and third order processes as shown in the following rate equation

$$\frac{dn(t)}{dt} = -k_1 n(t) - k_2 n(t)^2 - k_3 n(t)^3, \quad \text{(Eq-S16)}$$

where k_1 is the first order non-radiative trap-assisted recombination rate, k_2 is the second order radiative recombination rate constant and k_3 is the third order Auger recombination rate constant. k_1 is taken from the TCSPC measurements as shown in Table S5. k_3 is set as $10^{-29}\,cm^6 s^{-1}$ as obtained from literature[9] because the effect of k_3 becomes significant only when $n > 10^{18} cm^{-3}$.

Eq-S16 is first solved in terms of the experimentally observed quantity $x(t) = \Delta T/T$, which is the photoinduced change in THz transmission. $n(t)$ is proportional to $x(t)$ with the following relationship

$$n(t)^i = \varphi^{i-1} C^{i-1} x(t)^i, \quad \text{(Eq-S17)}$$

where i is the recombination order, φ is the photon-to-charge branching ratio, $C = \tilde{n}_0/x(0)$ is the proportionality factor between the absorbed photon density, $x(0)$ and the initial photon density, $\tilde{n}_0$ as shown below

$$\tilde{n}_0 = \frac{E\lambda\alpha(\lambda)}{hc\,A_{eff}}\left(1 - R_{Pump}\right), \quad \text{(Eq-S18)}$$

where d is the thickness of each sample. By substituting Eq-S17 into Eq-S16, Eq-S16 is rewritten as

$$\frac{dx(t)}{dt} = -k_{nonrad}x(t) - C\varphi R_{rad}x(t)^2 - C^2\varphi^2 R_{Auger}x(t)^3, \quad \text{(Eq-S19)}$$

The numerical solutions to this ODE are fitted globally to the decays using the least squares method across all fluences in order to extract φk_2. Given that $0 \le \varphi \le 1$, the values presented for k_2 in the main text are underestimated compared to the true intrinsic values.

In order to account for an initially spatially varying charge-carrier density (due to absorption following the Beer-Lambert law), the fitting algorithm takes into account an exponentially decaying charge-carrier profile created by the pump beam. This is done by dividing the sample into 30 equally thick slices and computing the decay function for each of these individually.

9. Device Measurements

<u>Current Density-Voltage Characteristics</u>
The current density-voltage (J-V) characteristics for perovskite solar cells were measured in air using a Keithley 2400 source meter under approximately 100 mW/cm^2 of AM1.5G irradiation generated by an ABET Sun 2000 Class A simulator. Before the device measurement, the intensity of the solar simulator was automatically measured by a KG5 filtered silicon reference cell (certified by Fraunhofer ISE) to calibrate the accurate power conversion efficiency (PCE). The illuminated active area was 0.25 cm^2 defined by black anodized metal masks. The *J-V* curves were taken from 1.2 V to -0.2 V and followed by the forward scan (from -0.2 V to 1.2 V), at a scan rate of 0.013 V/s in each direction. We measured 16 devices for each group to get the device performance statistic results, as shown in Figure 4a.

<u>External Quantum Efficiency</u>
The external quantum efficiency (EQE) of devices was measured using a Fourier transform photocurrent spectrometer in the FTIR setup mentioned in section 6. The active area of devices (0.25 cm^2) was illuminated by a tungsten halogen lamp. The accurate EQE value was calibrated by a Newport-calibrated reference silicon solar cell of a known EQE.

The short-circuit current from the EQE measurement was determined by the overlap integral of the

AM1.5 photon flux with the EQE.[10]

Urbach Energy

Urbach energy (E_u) measurements were taken on the EQE setup, using a near-infrared source and a 780 nm low-pass filter, such that the band edge could be taken at a high sensitivity. E_u was obtained by using the single exponential to fit to the data in the band edge region.

Supporting Data and Discussion

1. Evaporation Parameters

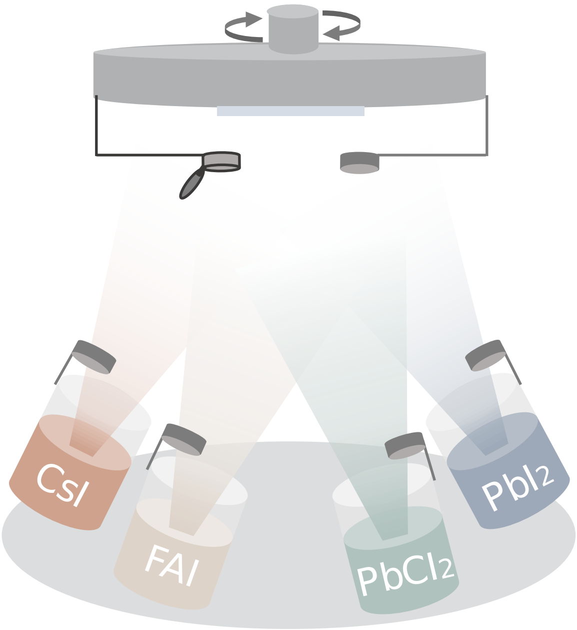

Figure S1 Simplified scheme of the four-source thermal co-evaporation method implemented in this study. Two QCMs close to the substrate are shuttered QCM (left) and open QCM (right).

Here, we start from monitoring the initial co-evaporation process. To accomplish this, we introduce one shuttered quartz crystal microbalance (QCM) in close proximity to the substrate position within the evaporator (as shown in Figure S1). By simultaneously opening the shuttered QCM and substrate shutters, the rate changes observed on the shuttered QCM can accurately reflect the actual deposition process occurring on the substrate. During the initial stages of the evaporation, we have observed that it takes a certain length of time for the rate to stabilize on a bare shuttered QCM (Figure S2a). Given previous knowledge that substrate types hugely influence the formed perovskite, we pre-coated the shuttered QCMs with some typical CTLs to reflect more accurately the growth of the perovskite on device relevant layers (Figure S2a). Here, we find that perovskites co-evaporated on CTLs need a longer stabilization time. The single-source evaporation is run to exclude that the observed stabilization time is caused by any single precursor (Figure S2b).

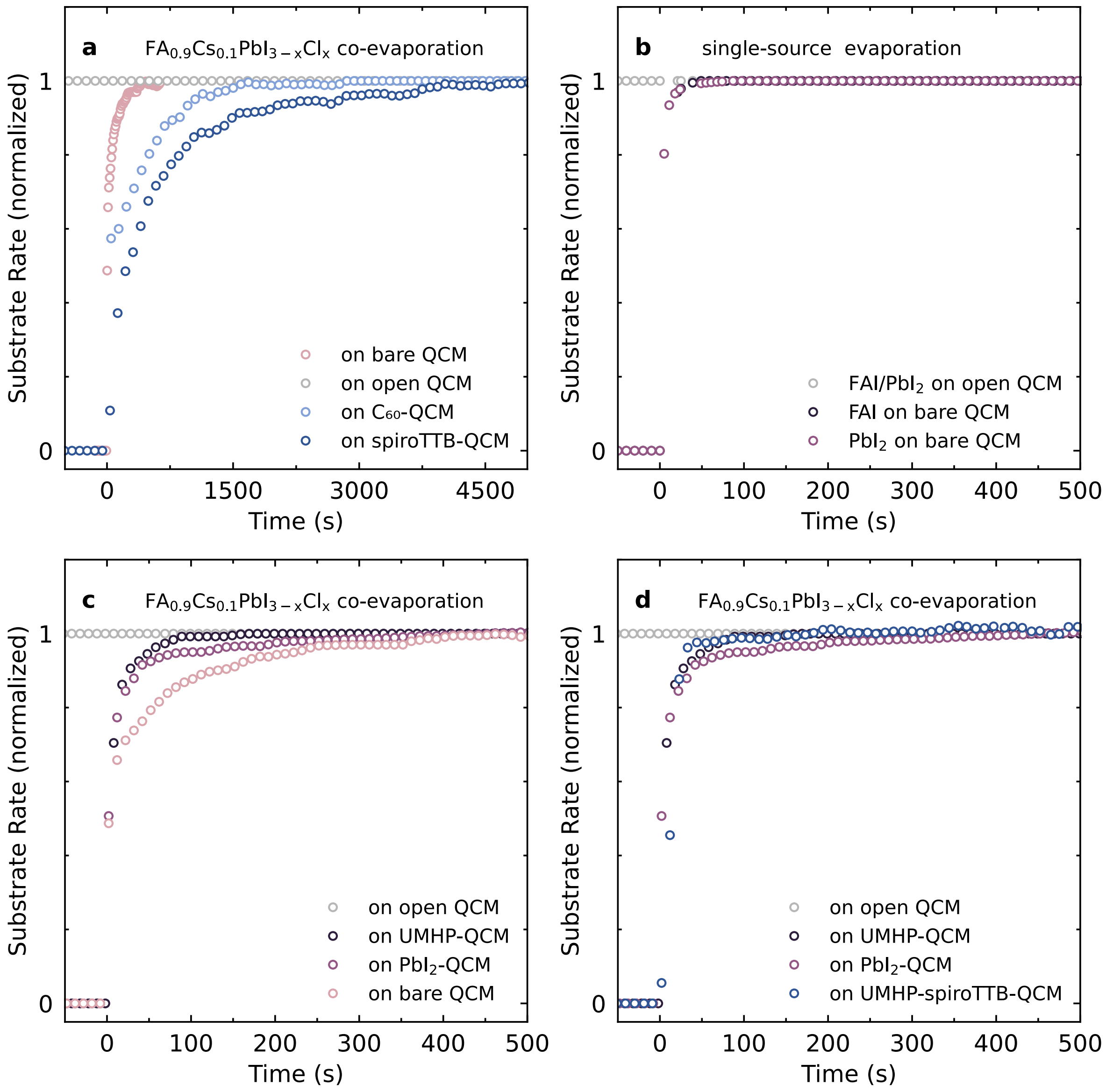

Figure S2 The substrate rate of initial co-evaporation process recorded by the QCMs. QCM shutter and substrate shutter are opened simultaneously at Time = 0s. (a) The initial process of $FA_{0.9}Cs_{0.1}PbI_{3-x}Cl_x$ co-evaporated on the open QCM, bare QCM (shuttered QCM without pre-coating), C_{60} pre-coated shuttered QCM and spiroTTB pre-coated shuttered QCM. (b) The initial process of FAI and PbI_2 single source deposited on the open QCM and bare shuttered QCM. (c) The initial process of $FA_xCs_{1-x}PbI_3$-Cl co-evaporated on the open QCM, ultra-thin MHP pre-coated shuttered QCM, PbI_2 pre-coated shuttered QCM and bare shuttered QCM. (d) The initial process of $FA_{0.9}Cs_{0.1}PbI_{3-x}Cl_x$ co-evaporated on the open QCM, ultra-thin MHP pre-coated shuttered QCM, PbI_2 pre-coated s-QCM and ultra-thin MHP-spiroTTB pre-coated shuttered QCM.

Table S1 Tooling factor of FAI on different substrates.

	**Tooling factor of FAI**
on ITO	0.1135
on PTAA	0.0686
on spiroTTB	0.1054
on Poly-TPD	0.1359
on C_{60}	0.1215
on NiO_x	0.0695
on SnO_2	0.0767
on PbI_2	0.1411
on ultra-thin MHP	0.1373

The tooling factor in the co-evaporation is termed as the ratio of the actual thickness of materials deposited on the substrate to the thickness displayed on the substrate QCM sensor. In the co-evaporation, the tooling factor is used to correct the real evaporation rate of precursors and therefore precisely control the film stoichiometry.

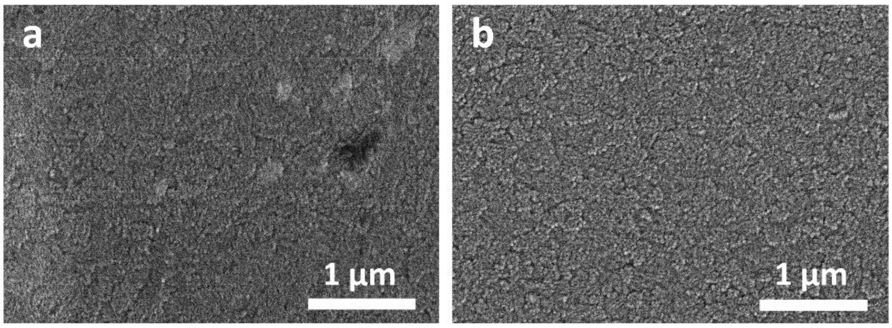

Figure S3 Top-view scanning electron microscopy images of the 4 nm (a) and 9 nm (b) PbI_2 deposited on ITO. Note that the 4-nm film is not thick enough to produce the continuous conformal PbI_2 layer. In contrast at 9-nm films are continuous and conformal.

2. Characterization of the templating layers

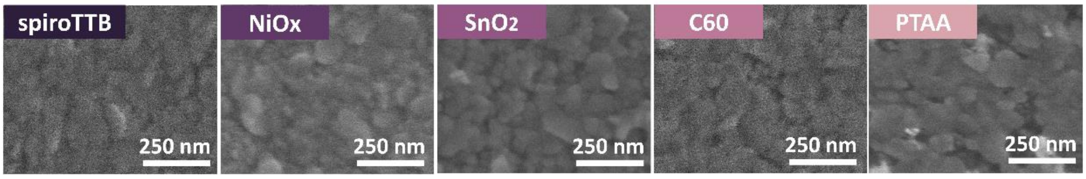

Figure S4 Top-view scanning electron microscopy images of the ultra-thin templating layers deposited on spiroTTB, NiOx, SnO_2, C_{60} and PTAA, respectively.

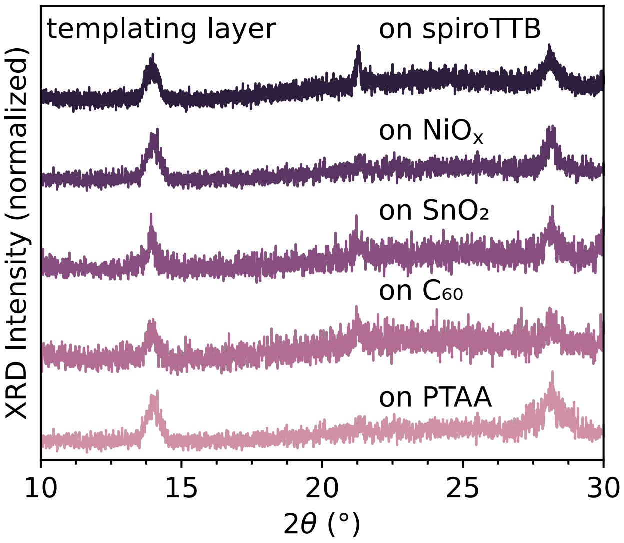

Figure S5 X-ray diffraction of the ultra-thin templating layers deposited on spiroTTB, NiO_x, SnO_2, C_{60} and PTAA, respectively.

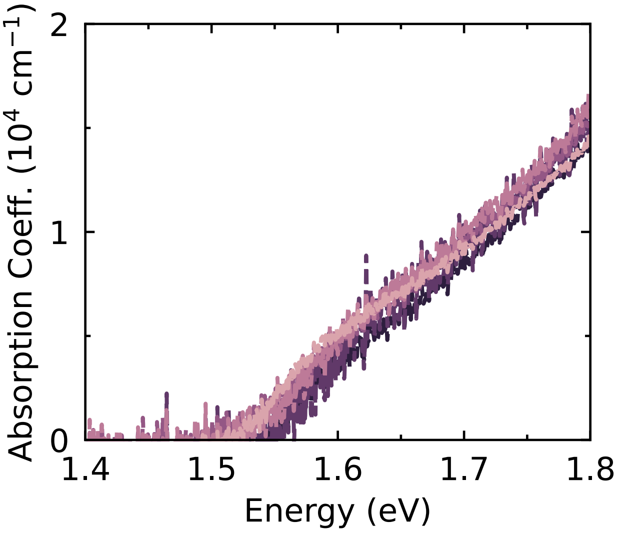

Figure S6 Absorption coefficient of the ultra-thin templating layers deposited on spiroTTB, NiO_x, SnO_2, C_{60} and PTAA, respectively. Note that the dynamic range and accuracy of the absolute value of the absorption coefficient are limited for these measurements owing to the ultrathin films being extremely thin (~15nm).

3. Film Morphology

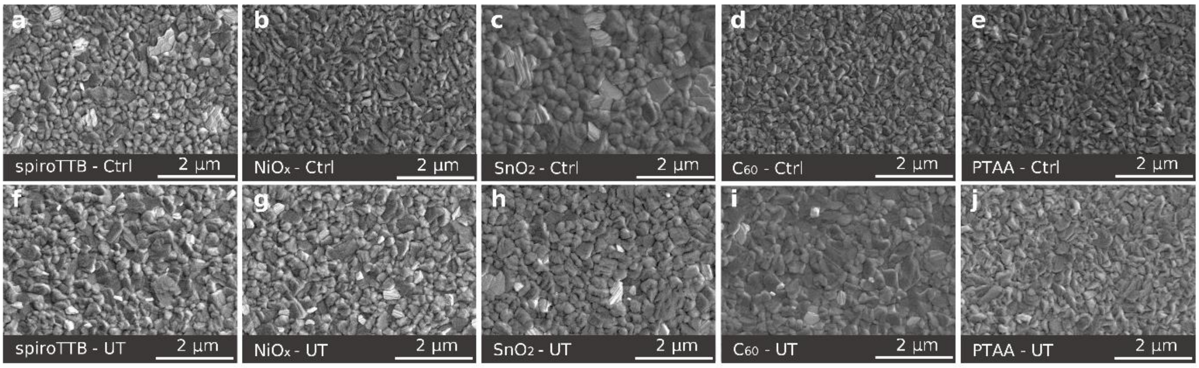

Figure S7 Perovskite film characterization. Top-view SEM images of Ctrl (a to e) and UT (f to j) films on spiroTTB, NiO_X, SnO_2, C_{60} and PTAA.

Table S2 Average thicknesses of Ctrl and UT films obtained from the cross-section SEM.

	**Ctrl Thickness (nm)**	**UT Thickness (nm)**
On spiroTTB	826.3±1.6	808.6±10.6
On NiO_x	847.8±13.1	808.1±20.3
On SnO_2	796.9±12.4	807.8±4.9
On C_{60}	767.8±13.9	803.1±8.2
On PTAA	837.6±9.8	802.0±4.9
On Textured Si	888.3±2.5	807.8±9.7

4. Absorption Measurements

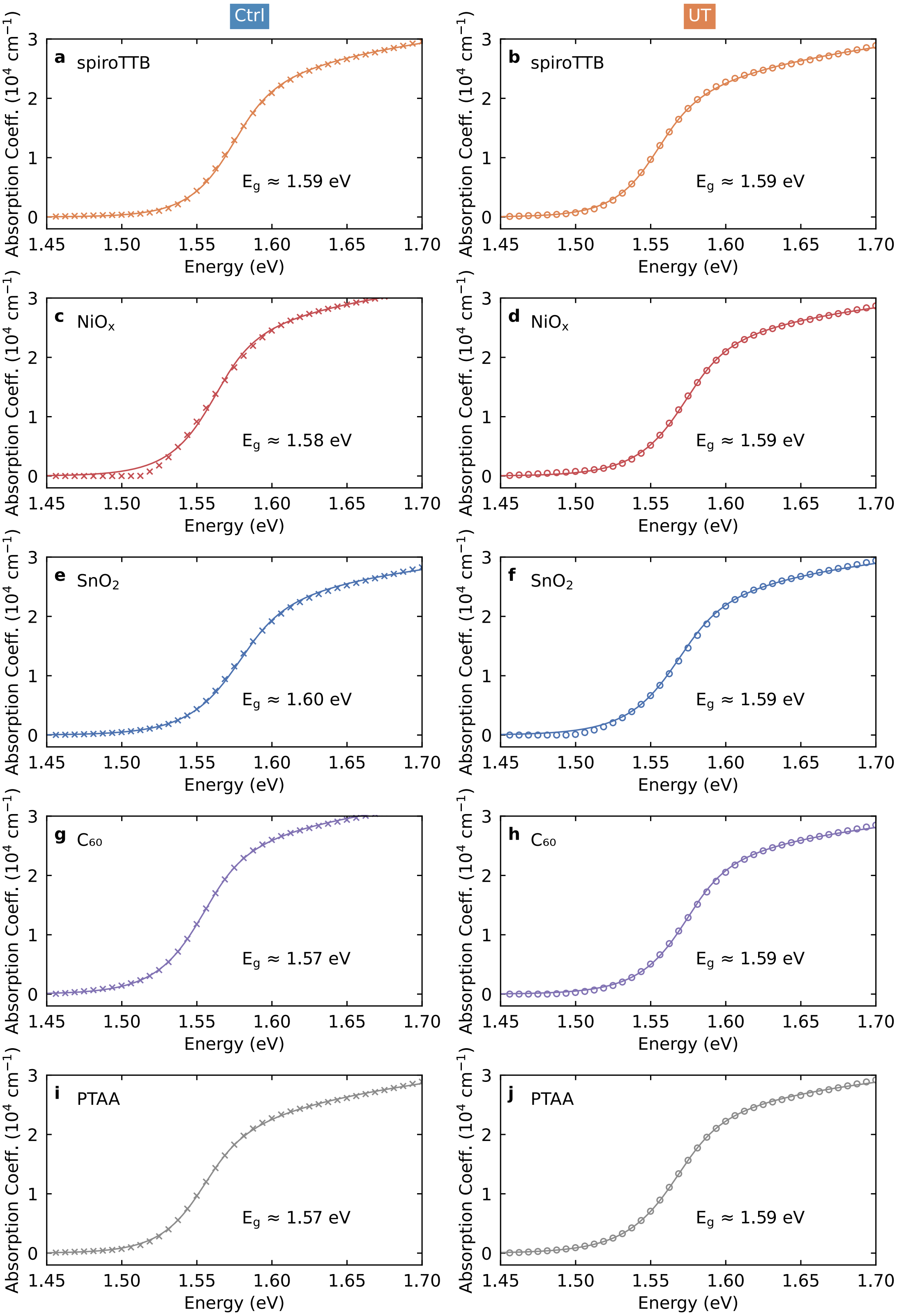

Figure S8 The absorption coefficient values of Ctrl and UT films on spiroTTB (a and b), NiO_X (c and d), SnO_2 (e and f), C_{60} (g and h) and PTAA (i and j). x and o symbols are experiment results. Solid lines are the Elliott fitting results.

Table S3 Parameters extracted from Elliott fits to the absorption onsets of Ctrl and UT films on different substrates. E_g representing the optical bandgap, E_B representing the exciton binding energy, γ representing the electron-phonon coupling, and Amp representing the amplitude of the spectrum.

	E_g (eV)		E_B (meV)		γ (meV)		Amp (a.u.)	
	**Ctrl**	**UT**	**Ctrl**	**UT**	**Ctrl**	**UT**	**Ctrl**	**UT**
On spiroTTB	1.59	1.59	6.3	6.2	19.8	18.5	78.0	78.9
On NiO_x	1.58	1.59	7.0	7.6	20.5	22.0	78.7	71.5
On SnO_2	1.60	1.59	7.5	7.5	22.9	23.1	71.4	72.6
On C_{60}	1.57	1.59	7.1	7.8	21.4	21.7	78.8	70.5
On PTAA	1.57	1.59	5.2	8.2	19.5	23.3	76.6	70.4

5. Crystallinity and Orientation Characterization

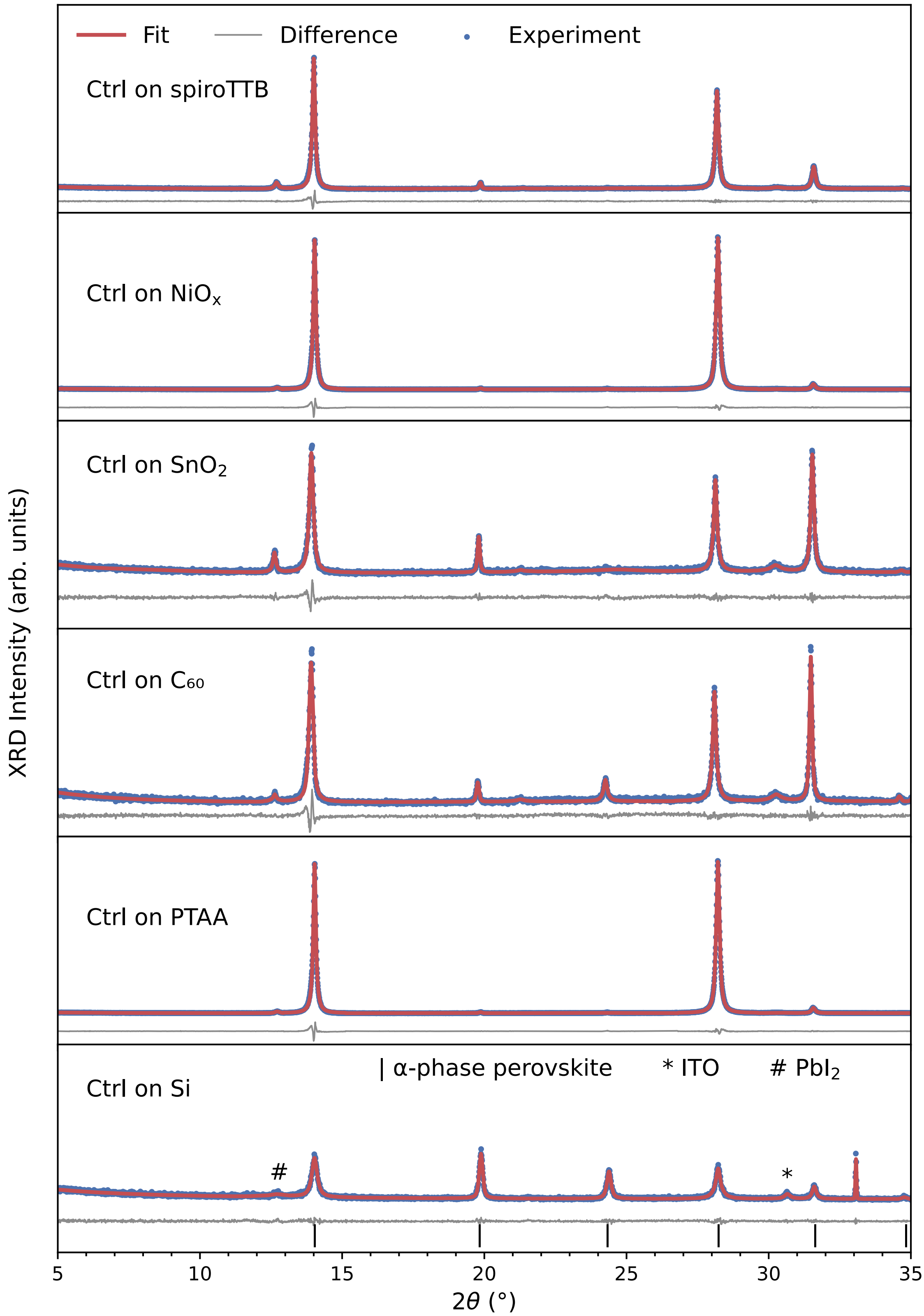

Figure S9 The XRD data (blue dots) fitted with a Rietveld fit (red line) and the difference (grey line) of Ctrl films on 6 different substrates. Tick marks below the bottom difference line show the peaks of α-phase perovskite. PbI_2 and ITO peaks are marked with # and *, separately.

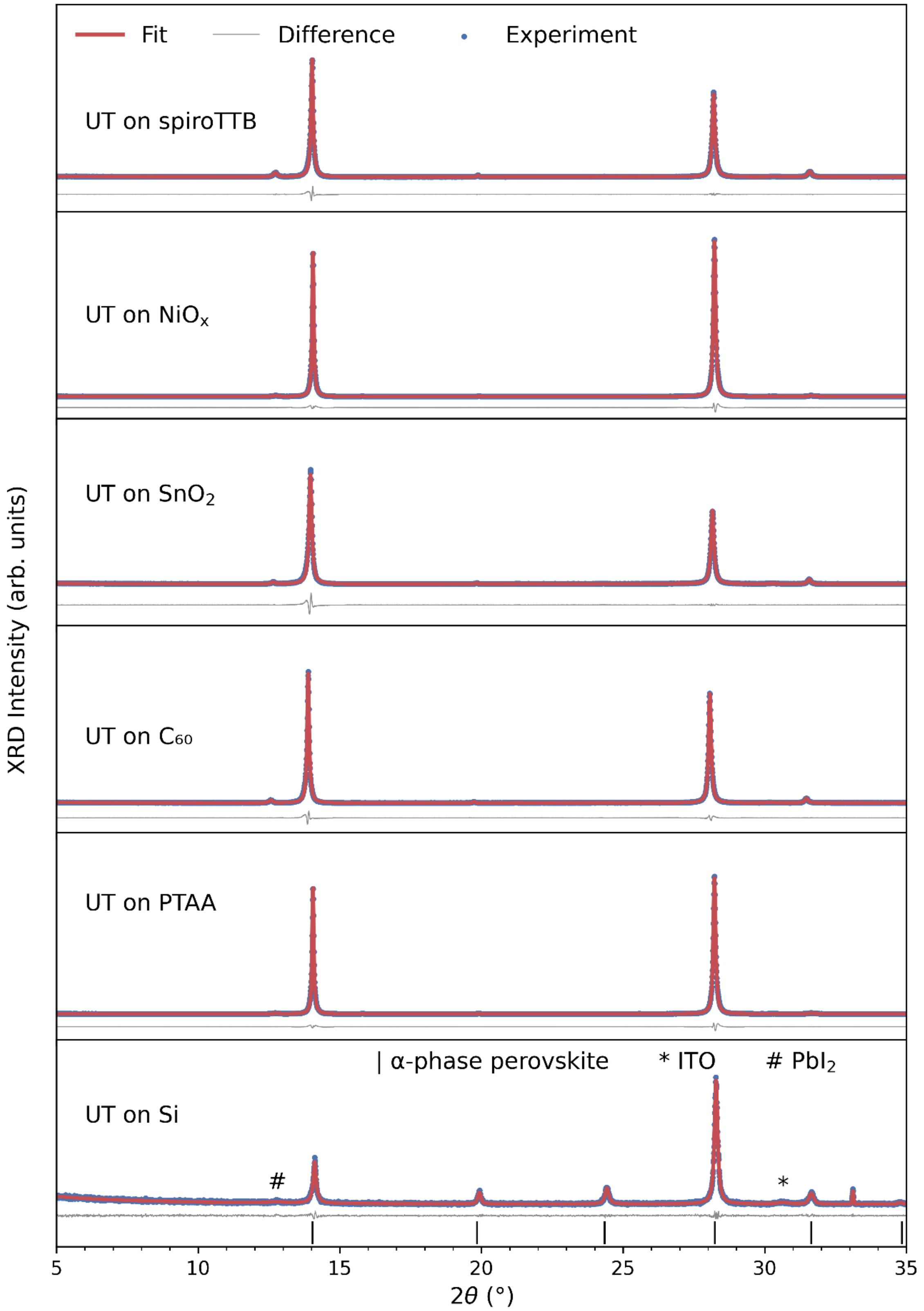

Figure S10 The XRD data (blue dots) fitted with a Rietveld fit (red line) and the difference (grey line) of UT films on 6 different substrates. Tick marks below the bottom difference line show the peaks of α-phase perovskite. PbI_2 and ITO peaks are marked with # and *, separately.

Table S4 Intensity and full width half maximum (FWHM) of (200) peak of Ctrl and UT films on various substrates.

	Scattering X-ray intensity (arb. units)		FWHM of (200) 2θ peak (degree)	
	Ctrl	UT	Ctrl	UT
On spiroTTB	4220.51	11987.63	0.1331	0.1227
On NiO_x	17094.67	105002.70	0.1403	0.1061
On SnO_2	879.00	8844.78	0.1397	0.1299
On C_{60}	922.35	18436.99	0.1409	0.1105
On PTAA	2524.18	48903.79	0.0954	0.1185
On Textured Si	292.57	1491.93	0.2029	0.1345

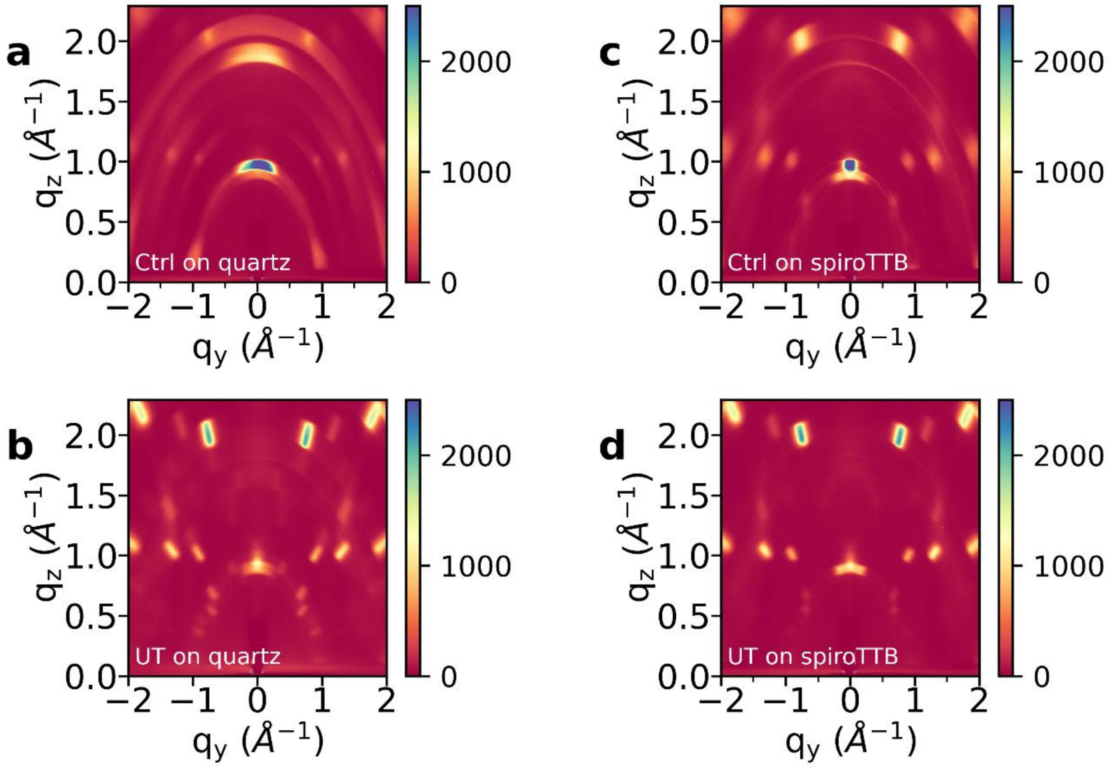

Figure S11 GIWAXS results of Ctrl and UT films on quartz (a and b), and spiroTTB (c and d), respectively. Figure 2c and 2d are replotted as Figure S11a and S11b for easier visualization.

6. Recombination Studies in Films

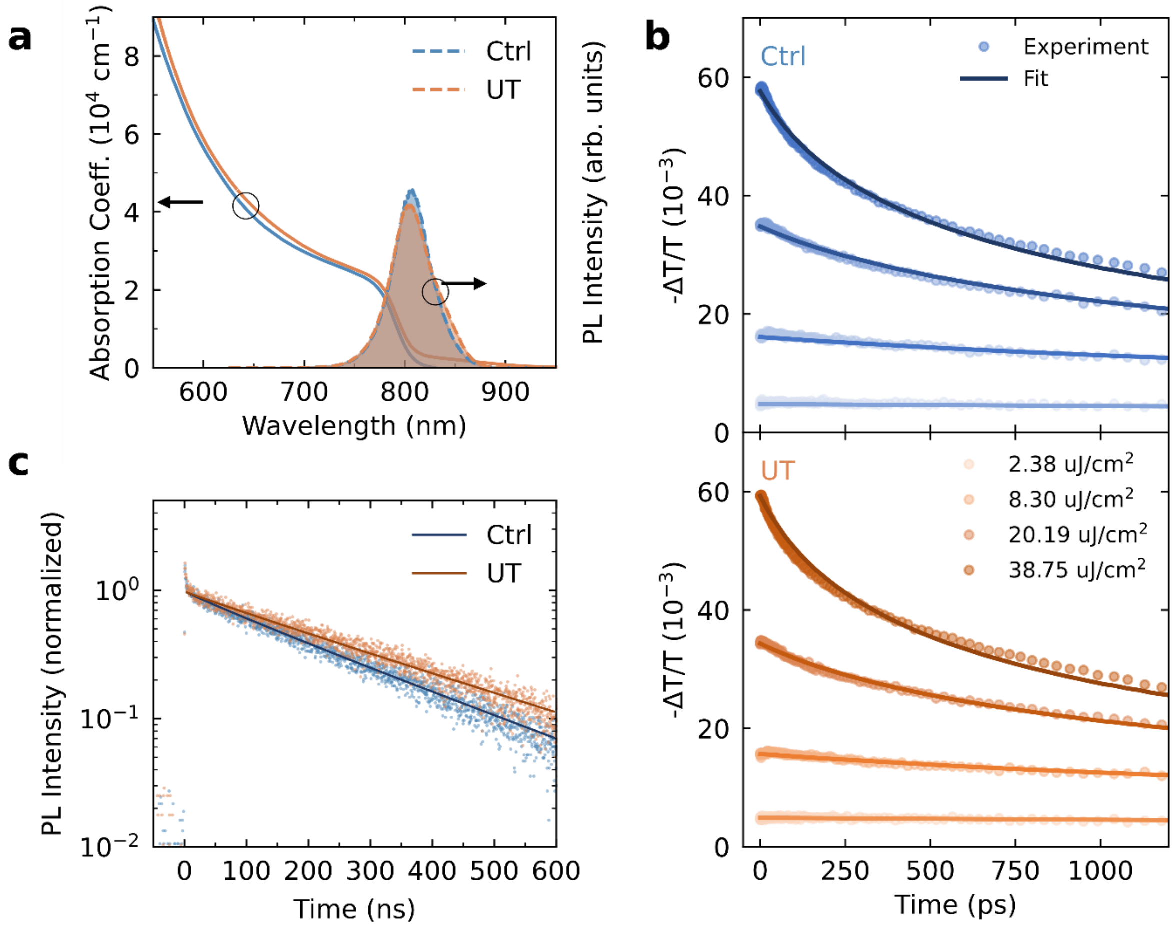

Figure S12 The optoelectronic properties of perovskite films. (a) Absorption coefficient and the unnormalized PL spectra (excited by the 470 nm laser) of Ctrl and UT films. (b) OPTP photoconductivity transients of Ctrl and UT films on quartz at various photoexcitation fluences. (c) PL dynamics of Ctrl and UT films on quartz (805 nm emission), at fluence 60 nJ/cm^2, fitted by stretched exponential without diffusion. The data is normalized to the value at 3 ns following the laser pulse allow for better visualization.

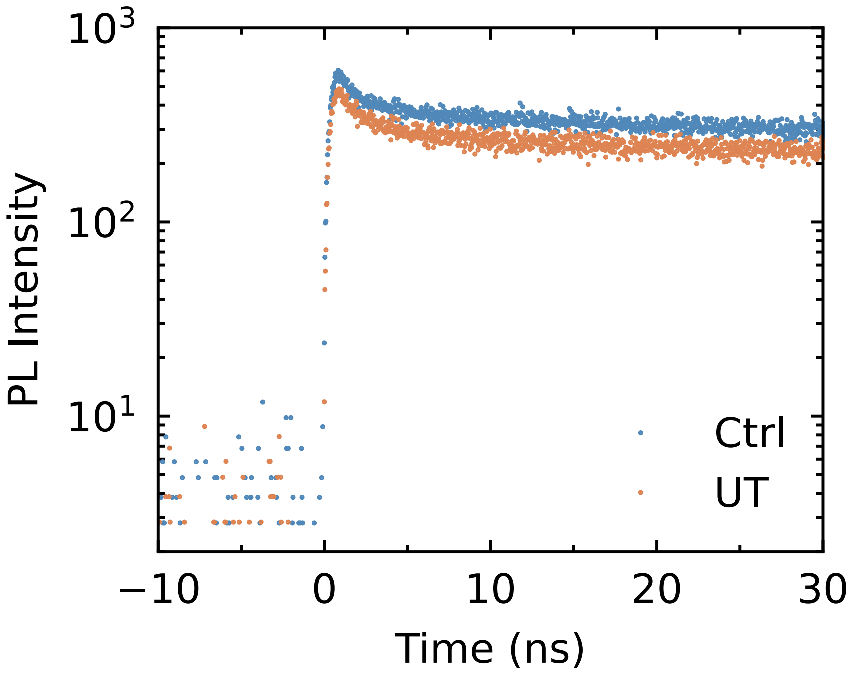

Figure S13 Unnormalized PL dynamics of Ctrl and UT films on quartz (805 nm emission), excited by the 470 nm laser at fluence 60 nJ/cm^2.

Table S5 Charge carrier mobility and second order recombination rate of Ctrl and UT films obtained from the OPTP measurement.

	THz mobility ($cm^2\ V^{-1}\ s^{-1}$)	k_2 [10^{-10}] ($cm^3\ s^{-1}$)
Ctrl	46.56±2.15	1.41±0.01
UT	43.62±1.53	1.37±0.01

Table S6 First-order non-radiative trap-mediated recombination parameters of Ctrl and UT films.

	τ_c (ns)	β	τ_{avg} (ns)	k_1 [10^6] (s^{-1})
Ctrl	214.35	0.94	220.50	≈2.27
UT	268.56	0.96	273.48	≈1.83

7. Device Optimization and Analysis

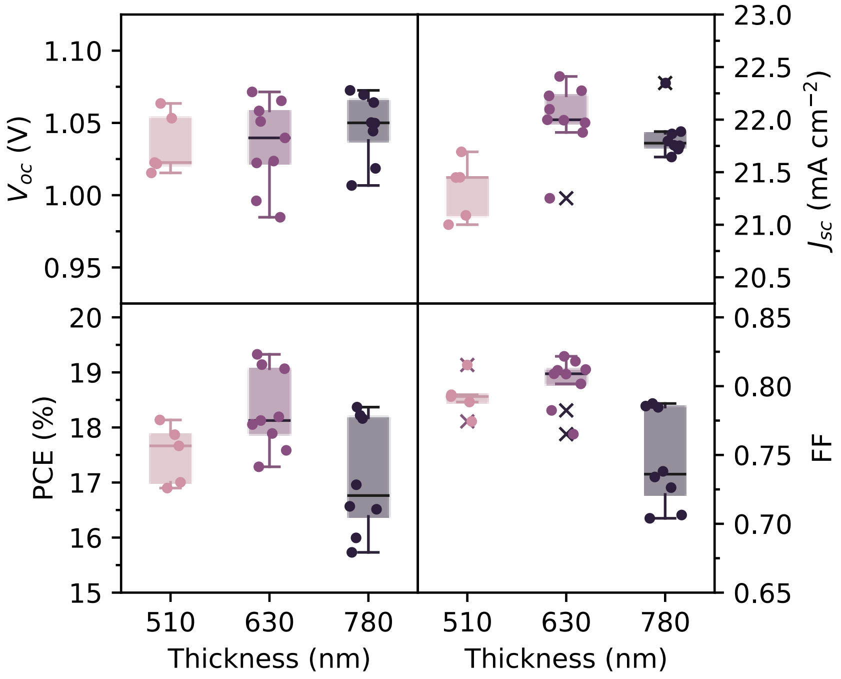

Figure S14 Statistical results of UT devices with the perovskite thickness of 510nm, 630nm, and 780nm, respectively.

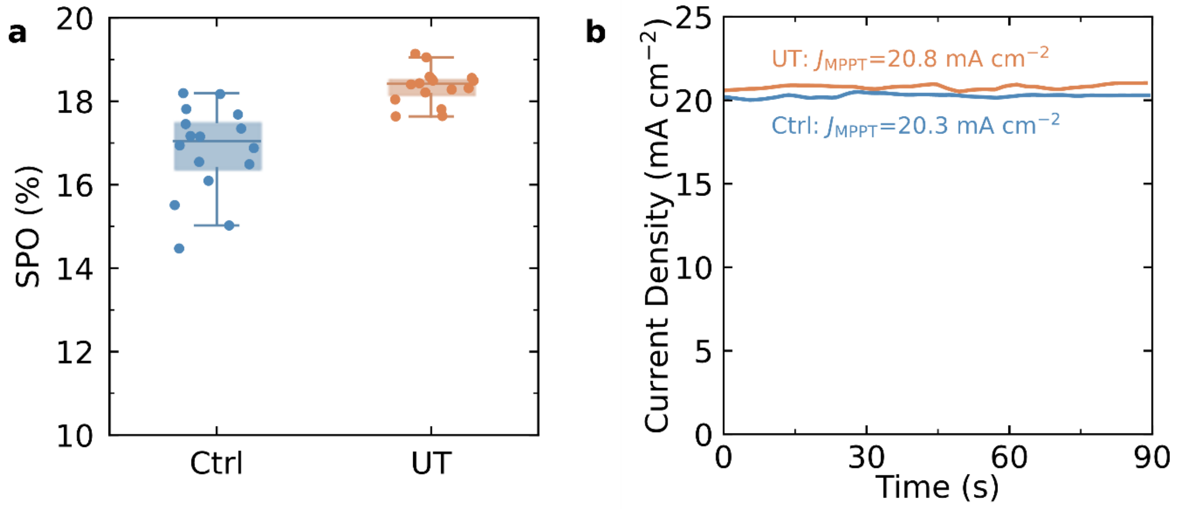

Figure S15 Steady-state results of Ctrl and UT devices. (a) Statistical steady-state efficiency (SPO) results. (b) Stabilized current density at maximum power point (J_{MPPT}). Previous studies have shown that measurements of SPO and J_{MPPT} can be more representative indicators of the true device efficiency since device kept at the constant bias is closer to the operation of a solar cell under load.[11]

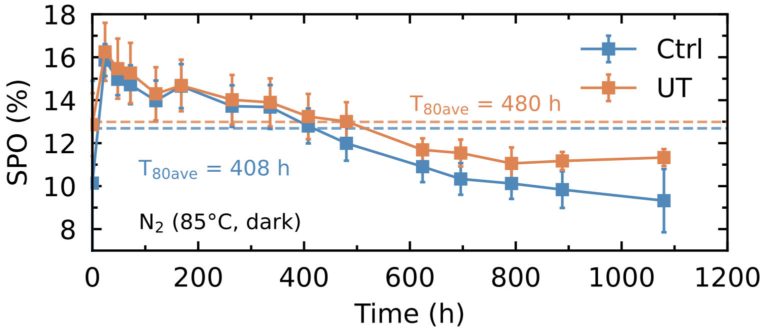

Figure S16 Evolution of SPOs of unencapsulated cells aged at 85°C in the dark N_2 atmosphere (three cells for each condition). The absorber layers are $FA_{0.9}Cs_{0.1}PbI_{3-x}Cl_x$ perovskite films with and without an ultra-thin MHP layer, labelled UT and Ctrl respectively. The device structure is ITO/spiroTTB/control or templated MHP films/C_{60}/Bathocuproine (BCP)/Au.

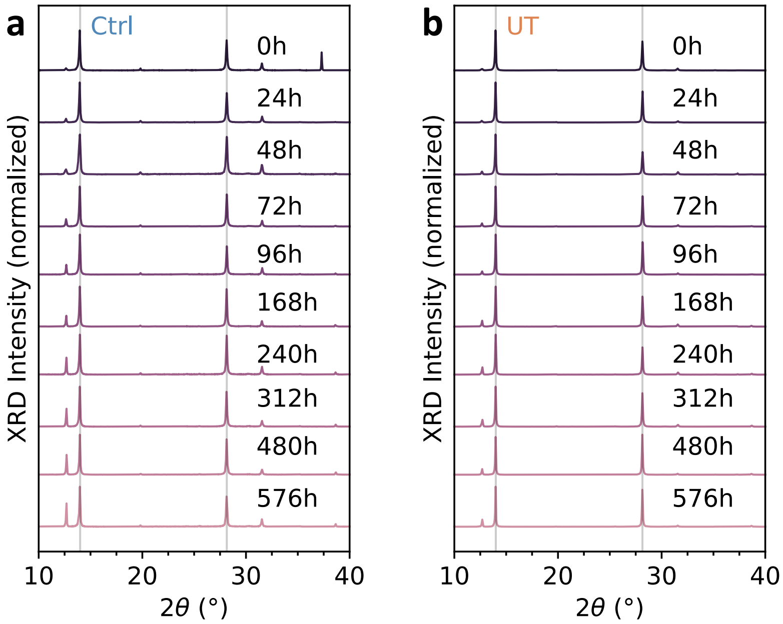

Figure S17 X-ray diffraction patterns of perovskite films aged at 85°C in the dark N_2 atmosphere. The absorber layers are $FA_{0.9}Cs_{0.1}PbI_{3-x}Cl_x$ perovskite films deposited on ITO/spiroTTB substrates with and without an ultra-thin MHP layer, labelled UT and Ctrl respectively. The Cu-Kα X-ray source (λ=1.54 Å) was set to 40 kV voltage and 40 mA current. The XRD patterns were corrected by shifting the 2θ-axis based on the ITO substrate reference peak at 2θ=30.3°.

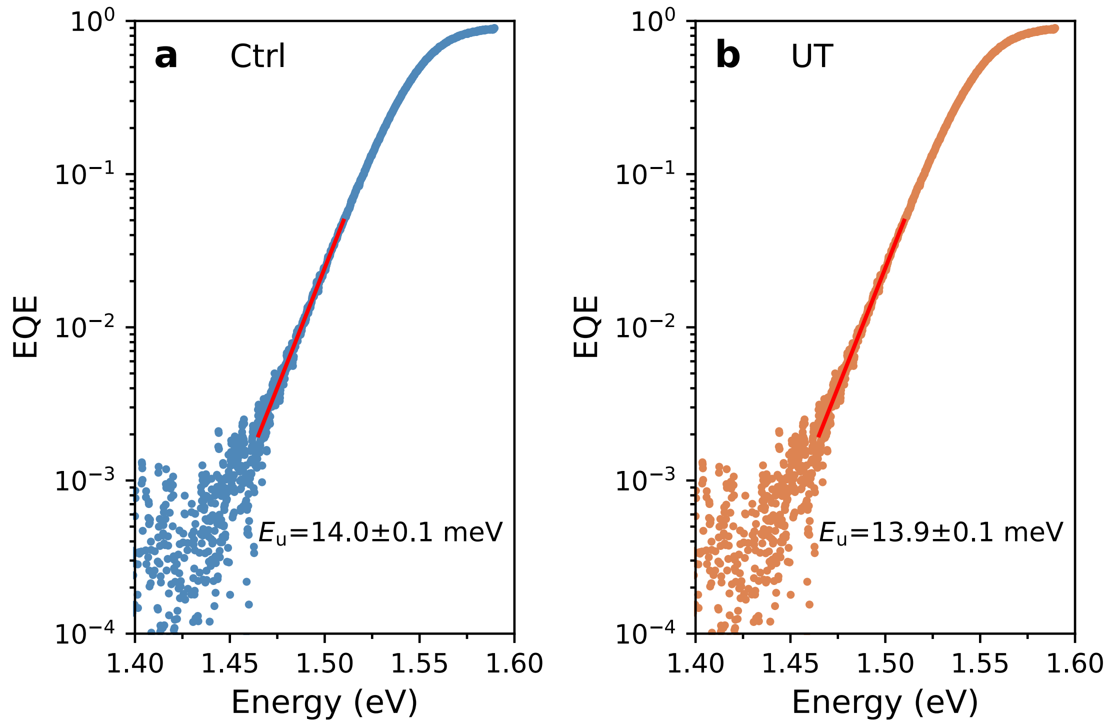

Figure S18 The Urbach energy extracted from the tail of EQE spectra of (a) Ctrl and (b) UT devices.

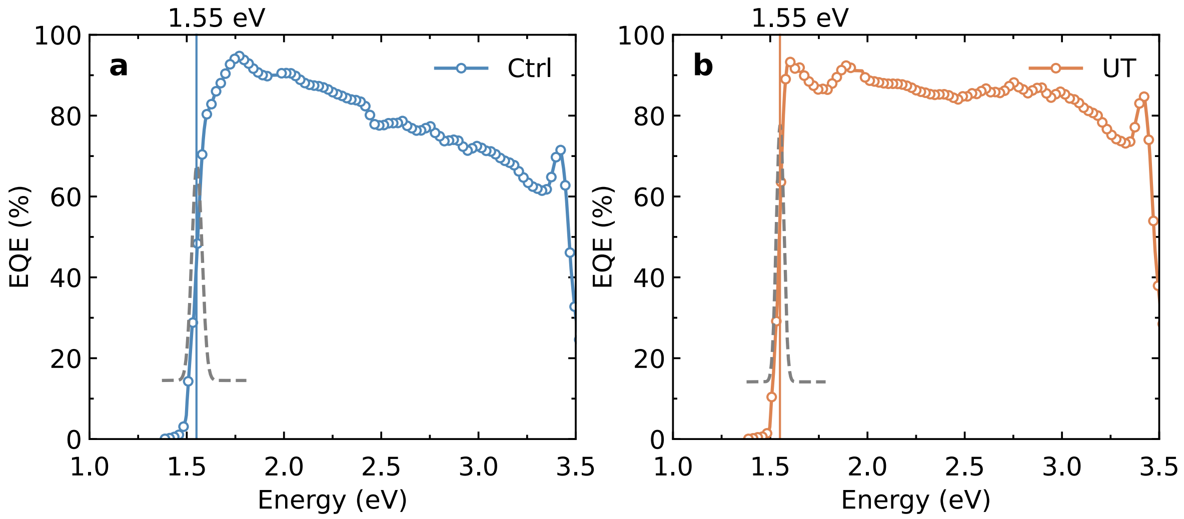

Figure S19 Bandgap values of Ctrl (a) and UT (b) devices extracted from the first derivative of the EQE (grey dashed lines) band edge as a function of photon energy.

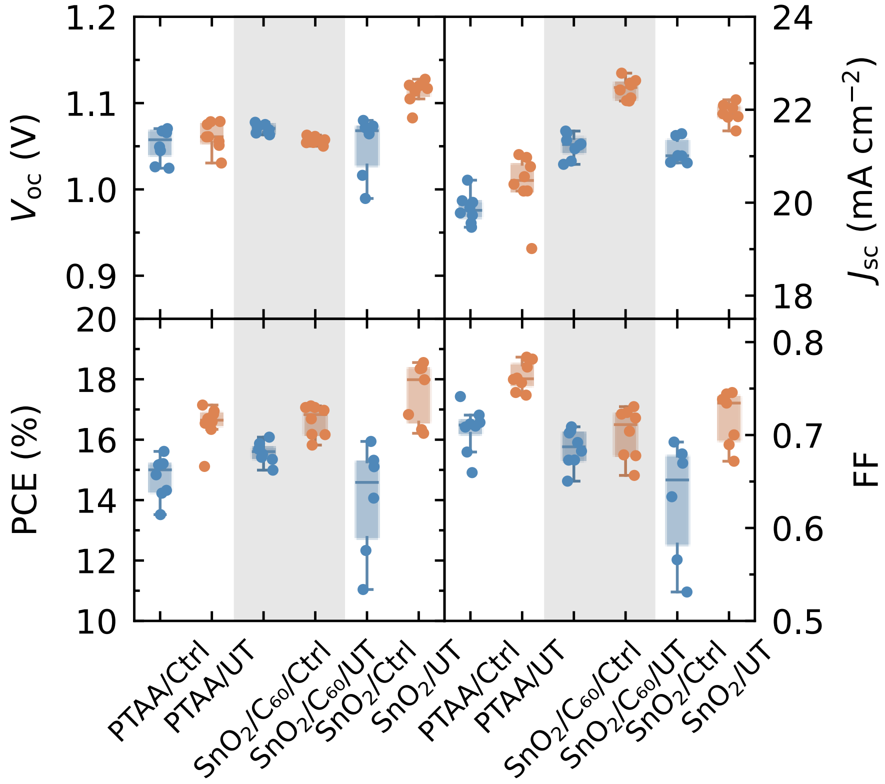

Figure S20 Statistical results of both p-i-n and n-i-p devices made by depositing Ctrl and UT films on organic (PTAA and C_{60}) and inorganic (SnO_2) substrates.

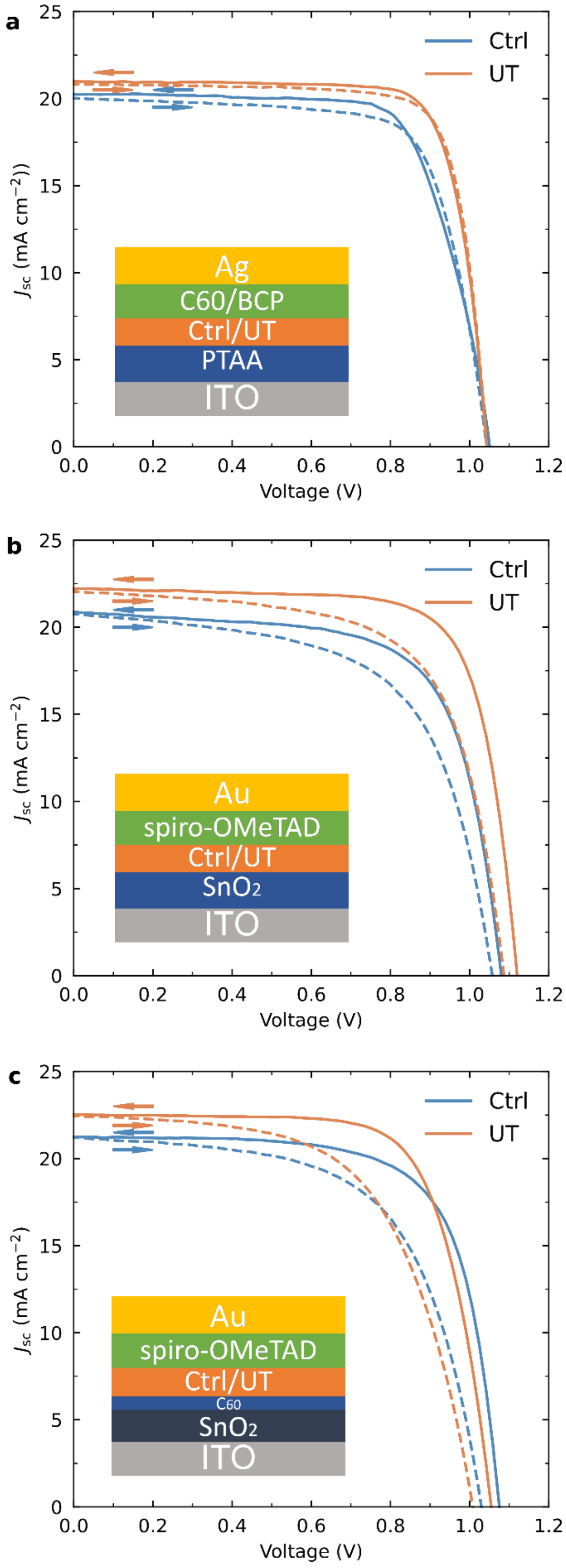

Figure S21 *J-V* characteristics and corresponding device architecture. (a) Ctrl and UT p-i-n devices with the HTL of PTAA. Ctrl and UT n-i-p devices with the ETL of SnO_2 (b) and C_{60} (c).

Table S7 Performance parameters for n-i-p solar cells fabricated from $FA_{0.9}Cs_{0.1}PbI_{3-x}Cl_x$ perovskite films with and without an ultra-thin MHP layer, labelled UT and Ctrl, measured under 1 sun simulated AM1.5G solar illumination. The values given in this table represent scans from reverse bias to short-circuit conditions at a scan rate of 0.013 V/s.

Device	J_{sc} (mA cm^{-2})	V_{oc} (V)	PCE (%)	FF	SPO (%)
SnO_2/Ctrl (champion)	21.5	1.07	15.9	0.69	13.6
SnO_2/Ctrl (average)	21.1±0.1	1.05±0.02	14.0±0.8	0.63±0.03	12.5±0.7
SnO_2/UT (champion)	22.2	1.12	18.6	0.75	17.2
SnO_2/UT (average)	21.9±0.1	1.11±0.01	17.5±0.4	0.72±0.01	16.2±0.5
C_{60}/Ctrl (champion)	21.2	1.08	16.1	0.70	16.1
C_{60}/Ctrl (average)	21.2±0.1	1.07±0.01	15.6±0.1	0.69±0.01	15.5±0.1
C_{60}/UT (champion)	22.5	1.05	17.1	0.72	16.9
C_{60}/UT (average)	22.4±0.1	1.06±0.01	16.6±0.2	0.70±0.01	16.3±0.2

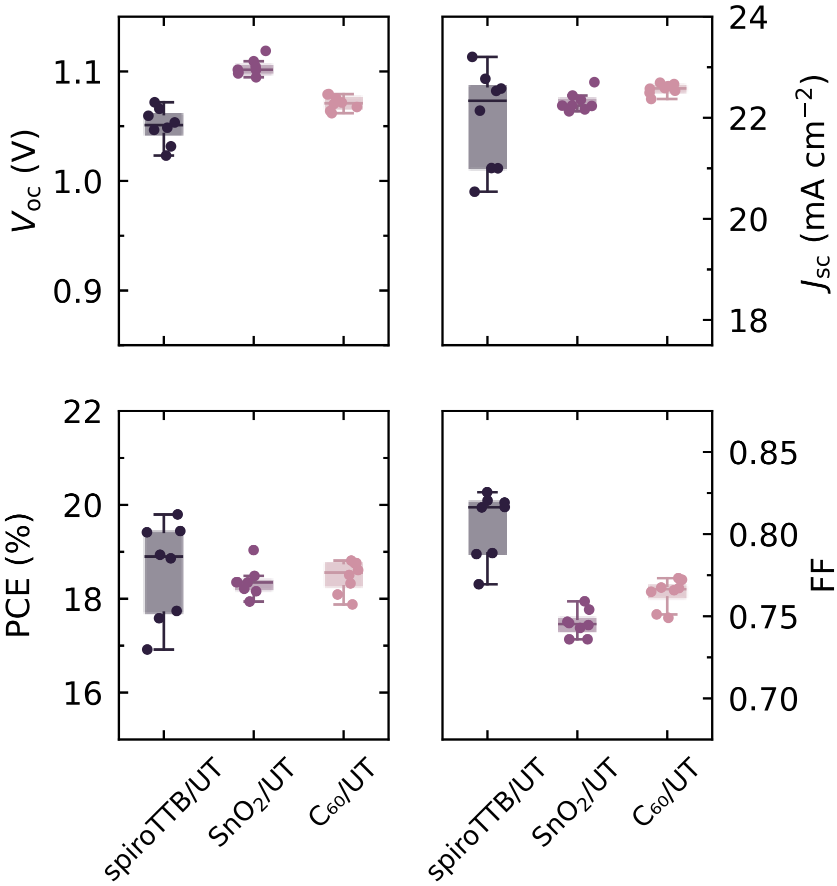

Figure S22 Statistical results of champion p-i-n devices with the HTL of spiroTTB and n-i-p devices with the ETLs of SnO_2 and C_{60} made in the same batch.

Table S8 Champion p-i-n and n-i-p device performance parameters for solar cells fabricated from UT films measured under 1 sun simulated AM1.5G solar illumination. The values given in this table represent scans from reverse bias to short-circuit conditions.

Device	J_{sc} (mA cm^{-2})	V_{oc} (V)	PCE (%)	FF	SPO (%)
spiroTTB/UT	22.8	1.06	19.8	0.82	19.8
SnO_2/UT	22.7	1.10	19.0	0.76	17.8
C_{60}/UT	22.6	1.08	18.8	0.77	18.6

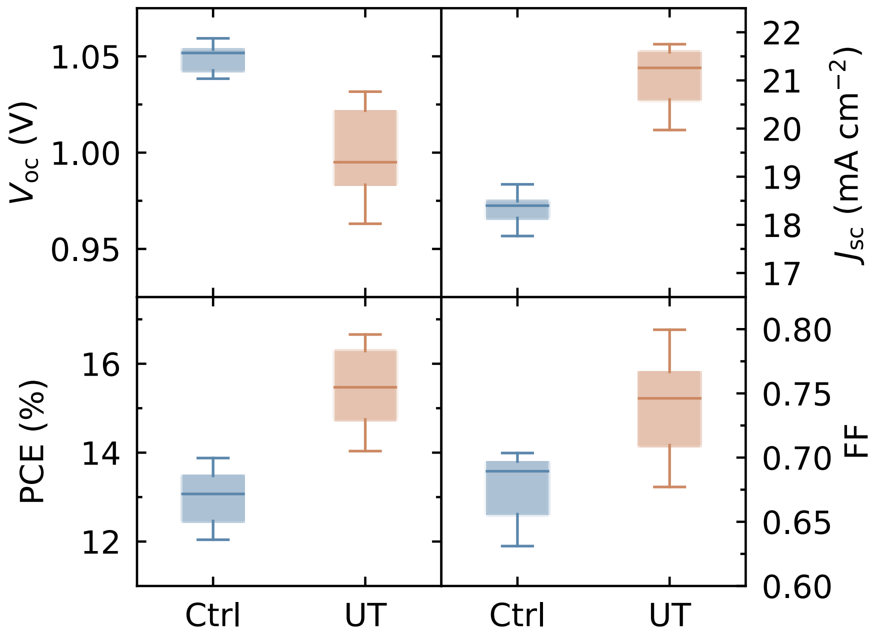

Figure S23 Statistical results of Ctrl and UT device parameters. The absorber layers are $FA_{0.9}Cs_{0.1}PbI_3$ perovskite films with and without an ultra-thin MHP layer, labelled UT and Ctrl respectively. The device structure is ITO/PTAA/control or templated MHP films/C_{60}/Bathocuproine (BCP)/Ag. The statistical results are taken from a total 7 pixels for each type.

References

(1) Davies, C. L.; Filip, M. R.; Patel, J. B.; Crothers, T. W.; Verdi, C.; Wright, A. D.; Milot, R. L.; Giustino, F.; Johnston, M. B.; Herz, L. M. Bimolecular Recombination in Methylammonium Lead Triiodide Perovskite is an Inverse Absorption Process. *Nat. Commun.* **2018,** *9* (1), 293.

(2) Elmestekawy, K. A.; Wright, A. D.; Lohmann, K. B.; Borchert, J.; Johnston, M. B.; Herz, L. M. Controlling Intrinsic Quantum Confinement in Formamidinium Lead Triiodide Perovskite through Cs Substitution. *ACS Nano* **2022,** *16* (6), 9640-9650.

(3) Tanaka, K.; Takahashi, T.; Ban, T.; Kondo, T.; Uchida, K.; Miura, N. Comparative Study on the Excitons in Lead-Halide-Based Perovskite-Type Crystals Ch3nh3pbbr3 Ch3nh3pbi3. *Solid State Commun.* **2003,** *127* (9), 619-623.

(4) Wright, A. D.; Verdi, C.; Milot, R. L.; Eperon, G. E.; Pérez-Osorio, M. A.; Snaith, H. J.; Giustino, F.; Johnston, M. B.; Herz, L. M. Electron-Phonon Coupling in Hybrid Lead Halide Perovskites. *Nat. Commun.* **2016,** *7* (1), 11755.

(5) de Quilettes, D. W.; Vorpahl, S. M.; Stranks, S. D.; Nagaoka, H.; Eperon, G. E.; Ziffer, M. E.; Snaith, H. J.; Ginger, D. S. Impact of Microstructure on Local Carrier Lifetime in Perovskite Solar Cells. *Science* **2015,** *348* (6235), 683-686.

(6) Richter, J. M.; Abdi-Jalebi, M.; Sadhanala, A.; Tabachnyk, M.; Rivett, J. P. H.; Pazos-Outón, L. M.; Gödel, K. C.; Price, M.; Deschler, F.; Friend, R. H. Enhancing Photoluminescence Yields in Lead Halide Perovskites by Photon Recycling and Light Out-Coupling. *Nat. Commun.* **2016,** *7* (1), 13941.

(7) Wehrenfennig, C.; Liu, M.; Snaith, H. J.; Johnston, M. B.; Herz, L. M. Charge-Carrier Dynamics in Vapour-Deposited Films of the Organolead Halide Perovskite $CH_3NH_3PbI_{3-x}Cl_x$. *Energy Environ. Sci.* **2014,** *7* (7), 2269-2275.

(8) Lohmann, K. B.; Motti, S. G.; Oliver, R. D. J.; Ramadan, A. J.; Sansom, H. C.; Yuan, Q.; Elmestekawy, K. A.; Patel, J. B.; Ball, J. M.; Herz, L. M.; Snaith, H. J.; Johnston, M. B. Solvent-Free Method for Defect Reduction and Improved Performance of p-i-n Vapor-Deposited Perovskite Solar Cells. *ACS Energy Lett.* **2022,** *7* (6), 1903-1911.

(9) Rehman, W.; McMeekin, D. P.; Patel, J. B.; Milot, R. L.; Johnston, M. B.; Snaith, H. J.; Herz, L. M. Photovoltaic Mixed-Cation Lead Mixed-Halide Perovskites: Links between Crystallinity, Photo-Stability and Electronic Properties. *Energy Environ. Sci.* **2017,** *10* (1), 361-369.

(10) Oliver, R. D. J.; Caprioglio, P.; Peña-Camargo, F.; Buizza, L. R. V.; Zu, F.; Ramadan, A. J.; Motti, S. G.; Mahesh, S.; McCarthy, M. M.; Warby, J. H.; Lin, Y.-H.; Koch, N.; Albrecht, S.; Herz, L. M.; Johnston, M. B.; Neher, D.; Stolterfoht, M.; Snaith, H. J. Understanding and Suppressing Non-Radiative Losses in Methylammonium-Free Wide-Bandgap Perovskite Solar Cells. *Energy Environ. Sci.* **2022,** *15* (2), 714-726.

(11) Patel, J. B.; Wong-Leung, J.; Van Reenen, S.; Sakai, N.; Wang, J. T. W.; Parrott, E. S.; Liu, M.; Snaith, H. J.; Herz, L. M.; Johnston, M. B. Influence of Interface Morphology on Hysteresis in Vapor-Deposited Perovskite Solar Cells. *Adv. Electron. Mater.* **2017,** *3* (2), 1600470.
